# Active-State Models of Ternary GPCR Complexes: Determinants of Selective Receptor-G-Protein Coupling

**DOI:** 10.1371/journal.pone.0067244

**Published:** 2013-06-24

**Authors:** Ralf C. Kling, Harald Lanig, Timothy Clark, Peter Gmeiner

**Affiliations:** 1 Department of Chemistry and Pharmacy, Emil Fischer Center, Friedrich Alexander University, Erlangen, Germany; 2 Department of Chemistry and Pharmacy, Computer Chemistry Center, Friedrich Alexander University, Erlangen, Germany; 3 Centre for Molecular Design, University of Portsmouth, King Henry Building, Portsmouth, United Kingdom; Medical School of Hannover, United States of America

## Abstract

Based on the recently described crystal structure of the β_2_ adrenergic receptor - G_s_-protein complex, we report the first molecular-dynamics simulations of ternary GPCR complexes designed to identify the selectivity determinants for receptor-G-protein binding. Long-term molecular dynamics simulations of agonist-bound β2AR-Gα_s_ and D2R-Gα_i_ complexes embedded in a hydrated bilayer environment and computational alanine-scanning mutagenesis identified distinct residues of the N-terminal region of intracellular loop 3 to be crucial for coupling selectivity. Within the G-protein, specific amino acids of the α5-helix, the C-terminus of the Gα-subunit and the regions around αN-β1 and α4-β6 were found to determine receptor recognition. Knowledge of these determinants of receptor-G-protein binding selectivity is essential for designing drugs that target specific receptor/G-protein combinations.

## Introduction

G-protein-coupled receptors (GPCRs) are proteins that enable signal transduction through biological membranes. The more than 800 GPCRs (including receptors for olfaction and taste) constitute the largest family of membrane proteins in the human genome [1]. GPCRs show pronounced structural variety in their binding pocket and can thus be activated by diverse extracellular signals including photon-induced changes in ligand conformation, small molecules, peptides and proteins [2]. Agonist binding causes structural rearrangements in the intracellular part of the receptor [3]–[9] that enable binding of a heterotrimeric G-protein and thus formation of the ternary complex consisting of agonist, receptor and G-protein [10]. The ternary complex induces the transmission of signals that activate both distinct physiological processes involving sensory impressions such as vision, smell and taste and neurological, cardiovascular, endocrine and reproductive functions that make GPCRs (and G-proteins) important targets for drug design [11].

After the structural characterization of the β_2_-adrenergic receptor (β2AR) bound to an antagonist [12], [13] and the first agonist-β2AR complexes [14], [15], the crystal structure of the β2AR together with its signal-transducing G_s_-protein was determined by Brian Kobilka and his team [16]. This spectacular work offers important structural insights into the nucleotide-free ternary signaling complex that will be important for the rational, structure-based design of biochemical or computational studies to investigate ternary complexes. The G-protein as an intracellular binding partner has been shown to be a prerequisite for capturing the fully-activated state of a GPCR in a crystal, since the recently determined structure of the β2AR bound to our agonist FAUC50 indicated a receptor conformation that was similar to the antagonist-bound form [14]. Only in the presence of a G-protein simulating nanobody [15] or the G-protein itself [16], could the rigid body movements described above be observed. Recently, NMR experiments investigating the dynamic behavior of β2AR emphasized the fundamental role of an intracellular binding partner in the stabilization of a fully-activated receptor conformation [17].

The crystal structure provides a physiological, atomistic template of a fully-activated G-protein-coupled receptor bound to and stabilizing a nucleotide-free G-protein. It represents a valuable template for homology modeling studies that explore high-affinity active-state binding sites of GPCR-G-protein complexes. Active-state homology models can be of great importance for identifying new agonist lead-structures, for example in docking campaigns [18]. Because many GPCRs can bind multiple G-protein-subtypes, models of individual receptor-G-protein complexes are needed to design functionally selective drugs inducing the activation of a particular G-protein to a higher extend than coupling to alternative G-protein subtypes.

Herein, we describe the first active-state homology model of a G-protein-coupled receptor in complex with its preferred G-protein based on the crystal structure of the β_2_-adrenergic receptor in complex with the G_s_-protein [16]. In order to identify the amino acids responsible for coupling selectivity between GPCRs and G-proteins, we examined the protein-protein interface of two different ternary complexes, the agonist-bound β2AR-Gα_s_ crystal structure and, based on the β2AR-Gα_s_-structure, two homology models of the dopaminergic D_2_ receptor (D2R), a drug target of particular interest for the treatment of neuropsychiatric disorders including Parkinson’s disease and schizophrenia [19], in complex with dopamine and Gα_i1_. We carried out one µs molecular-dynamics simulations in a hydrated bilayer built of dioleoylphosphatidylcholine-lipids (DOPC) for each, and investigated the receptor G-protein interface by computational alanine scanning mutagenesis.

## Results and Discussion

### Active-state Homology Models of D2R-Gα_i_

**Figure 1:**
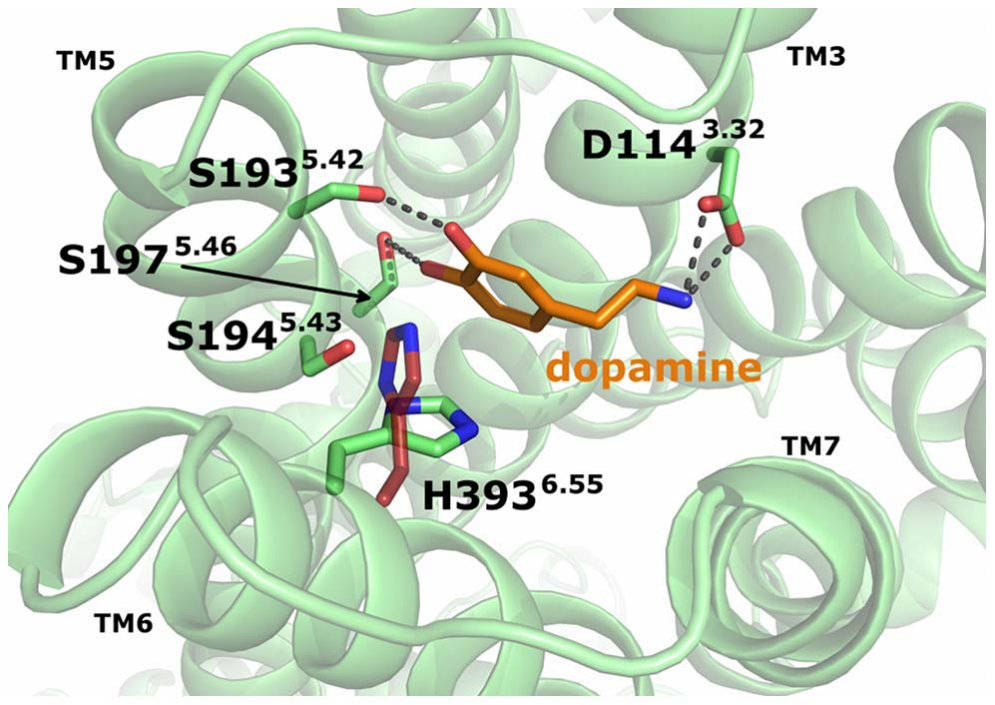
Initial conformation of dopamine in the D2R-Gα_i_-complexes. The backbone of D2R is shown as green ribbon, with important amino acids (indicated as green sticks) that stabilize the ligand dopamine in its initial conformation. Dopamine is represented as orange sticks and stabilized by ionic interactions to D114^3.32^ and hydrogen bonds to S193^5.42^ and S197^5.46^. The second conformation of residue H393^6.55^ is shown as red sticks.

According to Kobilka et al. [16], the “*active state of a GPCR can be defined as that conformation that couples to and stabilizes a nucleotide-free G-protein.*” We therefore used the crystal structure of the β2AR-Gα_s_-complex (PDB-ID: 3SN6) as a starting point for active-state homology models of D2R in complex with the nucleotide-free state of Gα_i_. We created alignments for the separated receptors and the G-proteins, combined them and subsequently started the modeling process using MODELLER 9v4. A more detailed description of the modeling process is provided in the Methods section. The models exhibited two different rotamer conformations of residue His393^6.55^ in D2R with the side chain of histidine pointing either to the extracellular or to the intracellular part of the receptor (Figure 1). His393^6.55^ has been shown to play a significant role in ligand binding and signaling bias at dopaminergic receptors [20]–[22] and that, in principal, both conformations are possible [23]. Therefore in the following studies, we decided to select two models of the D2R-Gα_i_-complex with both rotamer conformations of His393^6.55^, which are referred to in the following as D2^Up^R-Gα_i_ and D2^Down^R-Gα_i_. The physiological agonist dopamine was docked manually into D2^Up^R-Gα_i_ and D2^Down^R-Gα_i_ in a way that the positively charged ammonium head group forms a salt bridge to Asp114^3.32^ and that hydrogen bonds between the catechol moiety of dopamine and the side chains of Ser193^5.42^ and Ser197^5.46^ of D2R become feasible (Figure 1). These serine residues, Ser193^5.42^ and Ser197^5.46^, together with Ser194^5.43^, have been shown to be crucial for high-affinity catecholamine binding and for an effective receptor-G-protein coupling [24], [25].

Agonist binding of GPCRs leads to major structural changes within the receptors and the G-proteins that are consistent with the conformation of our active-state D2R-Gα_i_-complexes (Figure S1).

### Molecular-dynamics Simulations

Three ternary complexes, β2AR-BI167107-Gα_s_ and D2^Up^R/D2^Down^R-dopamine-Gα_i_, were successfully embedded into a hydrated DOPC-bilayer. We cleared a space for the initial insertion of the protein structures into the bilayer by removing DOPC-molecules from the bulk of the membrane (Figure S2a). A careful equilibration procedure was used to close the resulting gap between GPCRs and DOPC-residues (Figure S2b, c) without water molecules flooding this gap. The resulting complexes were subsequently submitted to molecular-dynamics (MD) simulations for one µs each, with the interior of the DOPC-bilayer remaining free of water throughout the simulations (Figure S3). The long simulation time of one µs for each complex was chosen to ensure the formation of sufficiently stable amino-acid contacts between the proteins in order to be able to elucidate amino acids that appear in the interface of GPCRs and G-proteins reliably.

All complexes remained very stable throughout the MD simulations showing low RMSD values for every member of the ternary complexes (Figure S4). As the G-proteins were not stabilized by membrane lipids, they showed higher atomic fluctuations than the receptor moieties (Figure S5). Substantial mobility was observed for the helical subunits of Gα_s_ and Gα_i_, Gα_s_AH and Gα_i_AH, which have previously been shown to become highly flexible in their nucleotide-free state [26], [27]. Comparing the atomic fluctuations of the two D2R-Gα_i_-complexes, we observed higher values for the D2^Up^R-Gα_i_-simulation (Figure S5), which were connected to a whole-body movement of Gα_i_ starting at the lower part of the α5-helix, but leaving the majority of α5 and its C-terminus unaffected (Figure S6a, b). The movement of Gα_i_ originates in the enhanced flexibility of open ends in the N-terminal IL3, which is mainly associated with the absence of the bulk of IL3 (Figure S5). This enhanced flexibility causes a loss of ionic interactions between residues from the N-terminal part of IL3 and residues from the area around α4-β6, which finally results in a displacement of Gα_i_ around helix α4 in the D2^Up^R-Gα_i_-simulation compared to the D2^Down^R-Gα_i_-complex. As this conformation appeared to be stable for the remainder of the simulation and did not lead to the separation of D2R and Gα_i_ (Figure S4, S7), we continued investigating both D2R-Gα_i_-complexes. Additionally, our data give no indication for any displacements of GPCRs and G-proteins other than the one described for the D2^Up^R-Gα_i_-complex.

**Figure 2:**
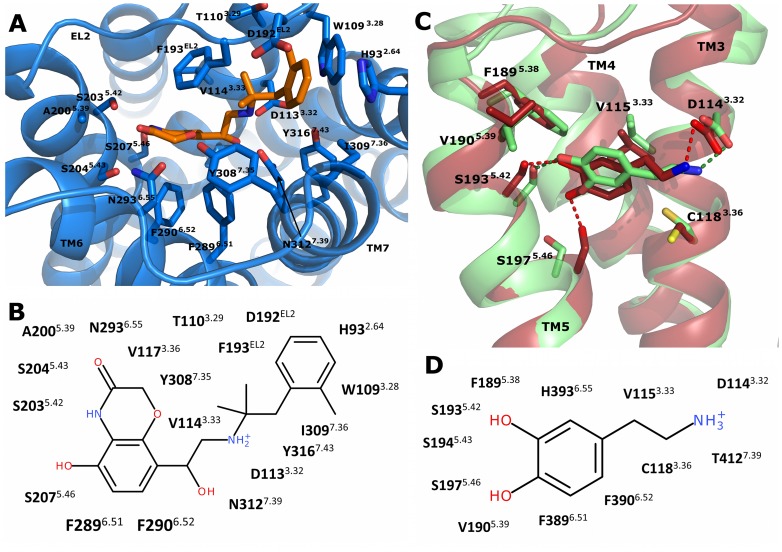
Characterization of the ligand binding pockets within the-simulation systems. (A) Extracellular view into the binding pocket of β2AR (blue ribbons). Residues involved in ligand binding are shown as blue sticks, whereas the ligand BI167107 is represented as orange sticks. (C) Side view into the binding pockets of the D2^Down/Up^R-models. Helices TM3, TM4 and TM5 are shown as ribbons (green: D2^Down^R; red: D2^Up^R), the other parts of the receptors are removed for clarity. Residues that stabilize dopamine in its binding pocket are represented as sticks. The different conformations of dopamine (green and red sticks) within the D2^Down^R- and D2^Up^R-simulations are depicted. (B, D) Schematic representation of interactions between the ligands BI167107 (B) and dopamine (D) and residues from β2AR and D2^Down/Up^R, respectively.

The agonists BI167107 and dopamine in the β2AR-Gα_s_-complex and in the D2R-Gα_i_-complexes, respectively, are largely enclosed in their binding pockets. In the β2AR-Gα_s_-complex, BI167107 maintained its interactions with residues of TM2, TM3, TM5, TM6 and TM7, most of which were already present in the crystal structure (Figure 2a, b). In the case of the D2R-Gα_i_-complexes, dopamine showed a different orientation of its catechol moiety within the binding pockets. Whereas only the *meta*-hydroxy group of dopamine formed a hydrogen bond to Ser193^5.42^ in the D2^Down^R-Gα_i_-complex, both, the *meta*- and *para*-hydroxy groups of dopamine were involved in the formation of hydrogen bonds to Ser193^5.42^ and Ser197^5.46^ of D2^Up^R, respectively (Figure 2c, d).

**Figure 3:**
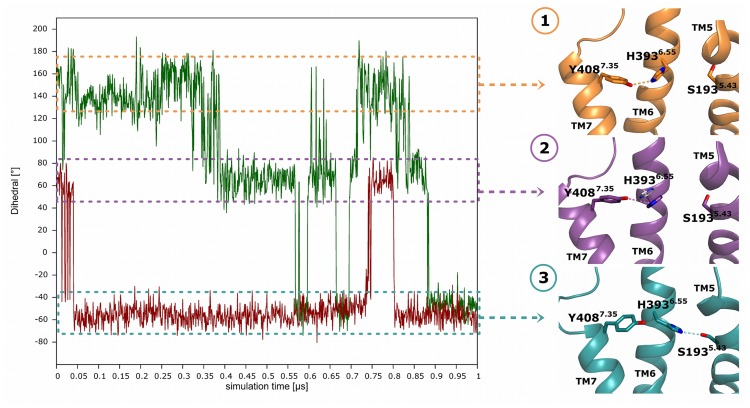
Dihedral angle of His393^6.55^ in the D2R-Gα_i_-complexes. On the left side of the figure, the dihedral angle of residue His393^6.55^ (atoms: C-CA-CB-CG) is depicted as green and red lines for the D2^Down^R-Gα_i_- and the D2^Up^R-Gα_i_-simulations, respectively. The right column shows representative snapshots taken from the D2R-Gα_i_-simulations and visualizes the interactions of residue His393^6.55^ with amino acids S193^5.43^ and Y408^7.35^ depending on its dihedral angle (orange: state 1; purple: state 2; dark-cyan: state 3). Helices 5, 6 and 7 are shown as ribbons, whereas the amino acids are represented as sticks. Additionally, state 2 shows the conformation of residue His^6.55^ taken from the crystal structure of the dopaminergic D_3_ receptor, as grey sticks.

This behavior may be associated with changes in the rotamer conformation of residue His393^6.55^ throughout the D2R-Gα_i_-simulations, where its side chain adopts three distinct dihedral angles, referred to as states 1, 2 and 3 (Figure 3). In state 1, the side chain of His393^6.55^ points towards the intracellular site of the receptor into the direction of TM7 (the initial conformation of the D2^Down^R-Gα_i_-complex), where it is stabilized by an interaction to residue Tyr408^7.35^ of upper TM7. State 2 shows the side chain of histidine pointing towards the extracellular part of the receptor (the initial conformation of the D2^Up^R-Gα_i_-complex and the one observed in the crystal structure of D3R), where it regains spatial proximity to Tyr408^7.35^ of TM7. The side chain is again oriented towards the intracellular site of the receptor in state 3, but now points in the direction of TM5, which enables a hydrogen bond to be formed to residue Ser193^5.43^. We assume that the dihedral angle of His393^6.55^ causes structural differences within the binding pocket of D2R, which lead to different conformations with respect to ligand binding. Structural connections between His^6.55^, Tyr^7.35^, TM5-serines and ligands that are able to discriminate between different downstream signaling pathways have been shown to be involved in biased signaling [23]. The agonist dopamine, which cannot cause functional selectivity, does not prevent the side chain of His393^6.55^ from cycling between its possible rotamer conformations. Sterically more demanding ligands may lock His393^6.55^ in one distinct rotamer conformation and thus trigger the activation of one distinct pathway. Therefore, further MD-simulations with selected ligands are necessary to elucidate the impact of His393^6.55^ on functionally selective signaling.

### The Receptor-G-protein Interface

Our µs MD-simulations were carried out in order to identify stable amino-acid contact sites between the receptors and their G-proteins that are maintained for long periods. Early experimental work, which focused on elucidating the interface between rhodopsin and its G-protein transducin using synthetic peptides that correspond to different regions of rhodopsin and transducin, identified the intracellular loops 2 and 3, the junction between TM7 and helix 8 of rhodopsin [28] and the area around α4-β6 and the C-terminal helix of transducin’s Gα subunit, Gα_t_ [29], as important contact sites between the two binding partners. These contact areas were further strengthened by a disulfide cross-linking study using the muscarinic M3 receptor and Gα_q_ [30]. A first structural glimpse of the amino acids involved in binding GPCRs to G-proteins was provided by crystallizing light-activated opsin together with a synthetic peptide (GαCT, residues ILENLKDCGLF) derived from the C-terminus of Gα_t_ [31]. By mutating the residues in GαCT into the corresponding amino acids of Gα_s_, we were able to delineate its interactions with β2AR [9]. Now, with the crystal structure of an entire ternary β2AR-Gα_s_ complex at hand, we have an excellent framework for investigating active-state models of structurally unknown ternary GPCR-complexes via computational methods.

**Figure 4:**
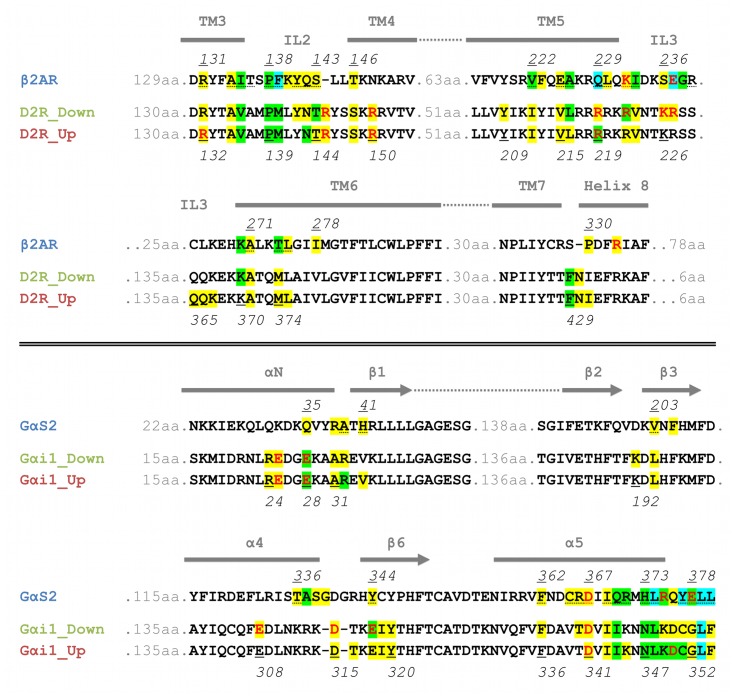
Alignment of the amino-acid contacts between receptors and G-proteins. Individual alignments for the receptors and the G-proteins are shown. A colored background indicates that the residue forms contacts to other amino acids (yellow: 1 or 2 contacts; green: 3 or 4 contacts; blue: at least 5 contacts). Red letters indicate residues involved in ionic interactions, whereas dotted underlines indicate contacts present in the crystal structure of β2AR-Gα_s_.

The trajectories of the MD simulations were therefore screened for amino-acid contacts between the receptors and the appropriate G-proteins. The receptor-G-protein interfaces are shown in Figure 4 as individual alignments for the receptors and for the G-proteins. Amino acids are highlighted in the alignment when at least one atom of an amino acid approaches at least one atom of another amino acid closer than 3.5 Å and when this interaction is found in more than 50% of the simulation. Detailed connection tables are provided in the (Table S1, S2, S3).

The receptor-G-protein interface of these fully-activated, nucleotide-free ternary complexes is comprised of homologous regions within the β2AR-Gα_s_-complex and the D2^Up/Down^R-Gα_i_-complexes. The amino-acid contacts within the two D2R-Gα_i_ simulations were found to be highly congruent, despite the differences concerning the displacement of Gα_i_ discussed above (Figure S6). GPCR contacts include the area around IL2, the N- and C-terminal parts of IL3 and the junction of TM7 and helix 8. The latter area only emerged as a contact region during the MD simulations and is not visible in the crystal structure of the β2AR-G_s_-complex. This observation underlines the importance of dynamic techniques such as MD simulation, which are not limited to a static snapshot of the protein. The G-protein contact regions consist of the αNβ1-loop, the area around β2-β3, the area around α4-β6 (with different distributions of the contact residues for the β2AR-Gα_s_- and the D2^Up/Down^R-Gα_i_-complexes) and the C-terminal α5-helix together with its C-terminus.

Additional information about the receptor-G-protein interfaces is given by highlighting the individual residues that appear in these interfaces with different colors that show the number of individual contacts from one residue to others: the darker the color (from yellow over green to blue) the more neighbors an amino acid has and the more important it is likely to be for receptor-G-protein coupling. Thus, the C-terminal domain of Gα, where high densities of tightly packed amino acids occur, can be assigned an outstanding role for complex stabilization and coupling selectivity arising from the G-protein. This is because the C-terminal α5-helix together with its extreme C-terminus is incorporated in the cavity formed by the outward movement of TM6 during receptor activation, which enables pronounced interactions with all of the contact regions of the GPCRs depicted. On the side of the receptors, we observed pronounced interactions for residues belonging to the areas around IL2 and the junction of the distal part of TM5 connected to the N-terminal part of IL3.

### Computational Alanine-scanning Mutagenesis

To elucidate the importance of each amino acid that appears in the interface between receptors and G-proteins, we carried out computational alanine-scanning mutagenesis of the β2AR-Gα_s_- and the D2^Up^R/D2^Down^R-Gα_i_-interfaces. This approach has been shown to be a valuable tool for estimating the contribution of individual amino acids to the stabilization of protein-protein interactions [32] and to be able to reproduce experimental investigations qualitatively [33]. We therefore used the MM-GBSA-method (Molecular Mechanics-Generalized Born Surface Area) [34], implemented in *MMPBSA.py* [35], to calculate the relative binding free energy changes (ΔΔG) between alanine-mutant complexes and the corresponding wild-type complexes in order to identify so-called hot-spot residues within the GPCR-G-protein interfaces that contribute to both coupling affinity and selectivity.

**Figure 5:**
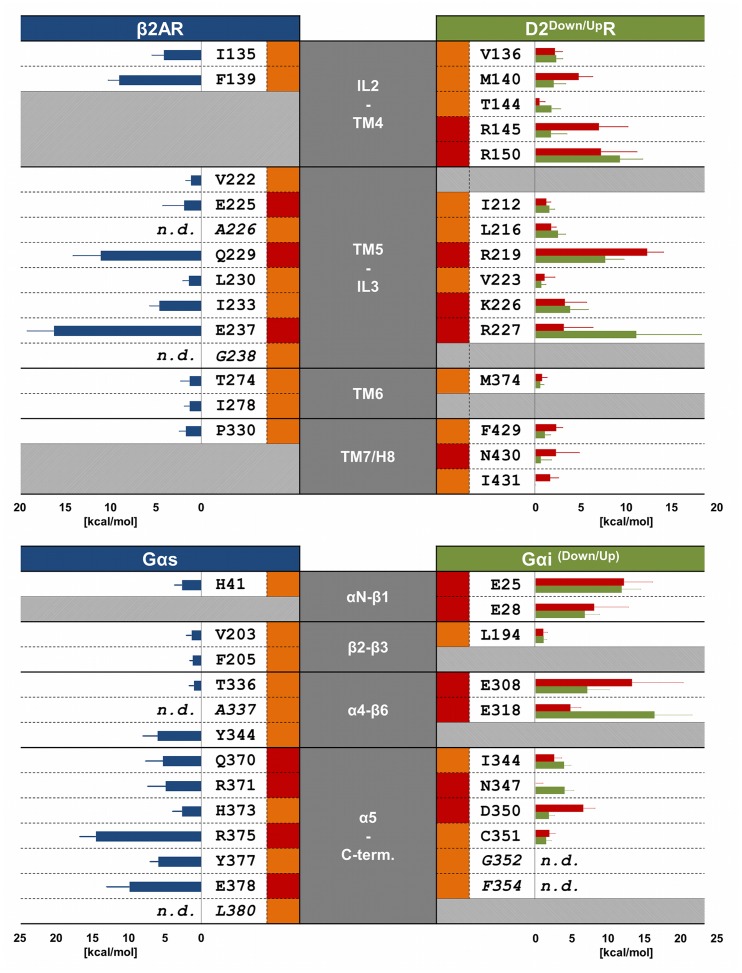
Summary of selectivity determining amino acids within theβ2AR-Gα_s_- and the D2R-Gα_i_-complexes and representative values of the alanine scanning mutagenesis. The grey columns in the middle refer to the regions within GPCRs and G-proteins, to which the mentioned amino acids belong. Amino acids in italic letters have not been mutated in the computational alanine scanning (n.d.). Blue, green and red bars show the binding free energy differences of the alanine scanning mutagenesis for the β2AR-Gα_s_ complex and the D2^Down^R-Gα_i_ and the D2^Up^R-Gα_i_-complexes, respectively. The orange and red rectangles besides the amino acids correspond to hydrophobic or polar interactions to other residues, respectively.

In a first step, we omitted water and membrane molecules and calculated the binding free energies (ΔG) of the β2AR-Gα_s_- and the D2^Up^R/D2^Down^R-Gα_i_-interfaces using the GBSA-method within *MMPBSA.py* in order to prove that the complex is energetically favorable and that the energy values remain generally consistent over the time scales investigated. Conformationally stable time periods within the three ternary complex trajectories were identified based on RMS deviations (Figure S4) and used to generate the required trajectories for the receptor- and the G-protein-parts with intervals of 500 ps between snapshots. Our calculations showed consistently negative ΔG-values for the systems on the time scales investigated, which indicates energetically favorable interactions between receptors and G-proteins (Figure S8). We subsequently performed computational alanine-scanning mutagenesis for the amino acid residues within the receptor-G-protein interfaces of β2AR-Gα_s_- and the D2^Up^R/D2^Down^R-Gα_i_-complexes that are highlighted in Figure 4, except for alanine-, glycine- and the C-terminal residues L380 and F354 from Gα_s_ and Gα_i_, respectively. In cases where only one amino acid of the D2^Up^R/D2^Down^R-Gα_i_-complexes constitutes a contact residue, we nevertheless performed alanine scanning on both amino acids. Important results of the alanine scan are shown in Figure 5, the complete results are provided in the (Table S4). In general, a positive value for the binding free energy change (ΔΔG) is associated with an amino acid that contributes to stabilizing the ternary complex, and vice versa.

**Figure 6:**
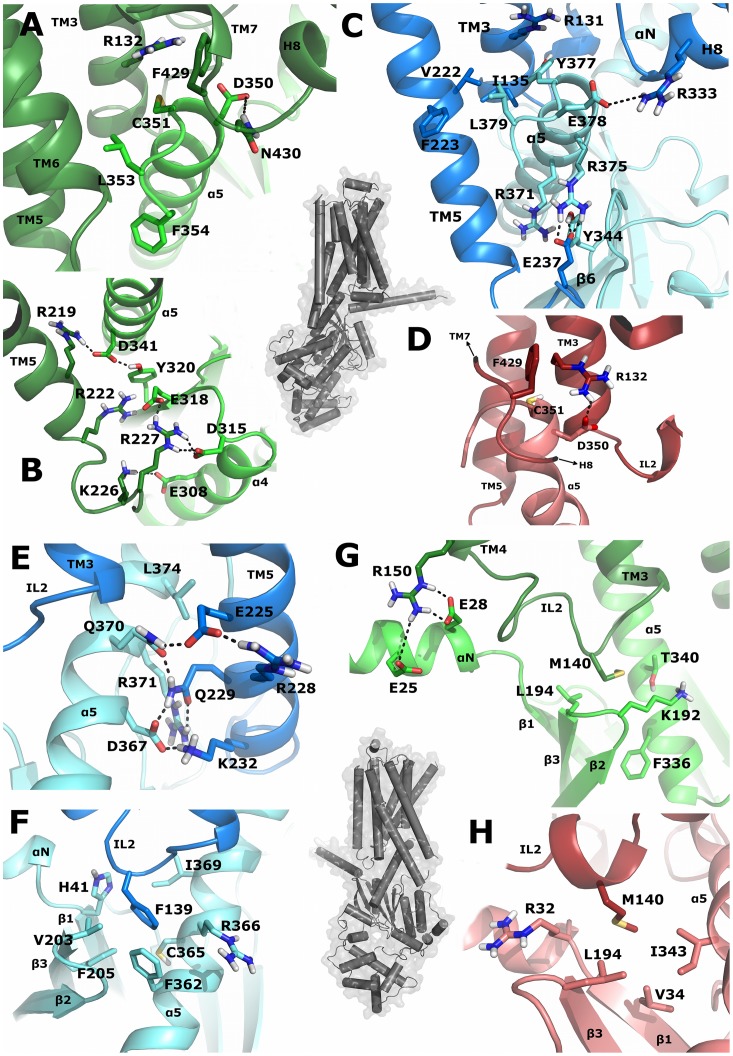
Crucial interactions between receptors and G-proteins. Residues in the receptor-G-protein interfaces of the simulation systems are shown as sticks. The receptors (dark-blue: β2AR, dark-green: D2^Down^R, dark-red: D2^Up^R) and the G-proteins (light-blue: Gα_s_, light-green: Gα_i_^Down^, light-red: Gα_i_^Up^) are represented as ribbons. Overview structures of the β2AR- Gα_s_-complex are indicated as grey tubes. The yellow rectangles point to the areas of the complexes, from which snapshots from MD simulations are visualized. (A) Specific interactions of amino acids from the C-terminus of Gα_i_ with residues from D2^Down^R are shown. (B) Ionic interactions between positively charged amino acids from IL3 of D2^Down^R and negatively charged amino acids of α5 and α4-β6 are depicted. (C); Crucial interactions of amino acids from the C-terminal part of Gα_s_ with residues from β2AR are shown. (D) The salt bridge between R132 of D2^Up^R and D350 of Gα_i_ is visualized. (E) Q229 of β2AR is a crucial amino acid within a hydrogen bond network formed between β2AR and Gα_s_. (F) F139 of β2AR shows pronounced hydrophobic interactions with residues from Gα_s_. (G, H) Interacting amino acids of IL2 from D2^Down^R (G) and D2^Up^R (H) with multiple domains of Gα_i_ are depicted. Residue M140 is differently stabilized within the D2^Down^R and D2^Up^R simulations.

For the β2AR-Gα_s_-system, we found that residues R131, I135, F139, Q229, K232, I233, E237, K270 and R333 from β2AR and H41, Y344, D367, I369, Q370, R371, H373, L374, R375, Y477, E378 and L379 from Gα_s_ stabilize the receptor-G-protein interface. Among these residues, F139 from IL2, Q229 and E237 from TM5-IL3 and R333 from helix 8 have been found to be of major importance for β2AR, whereas D367 and R375 from α5 and E378 from the C-terminus of the Gα-subunit are important for Gα_s_. F139 interacts tightly with a hydrophobic pocket comprised of residues H41, V203, F205, F362, C365, R366 and I369 from Gα_s_ (Figure 6f, Table S1) and thus stabilizes the interface of IL2, αN-β1, β2-β3 and α5. It has been shown that mutating F139 to alanine in β2AR prevents activation of adenylyl cyclase by Gα_s_ and that, in general, a bulky amino acid is necessary in this position for effective receptor-G-protein coupling [36]. Residue Q229 from the N-terminal IL3 forms the center of an extended hydrogen-bond network to residues D367, Q370 and R371 of the α5-helix of Gα_s_ and K232 from TM5 of β2AR (Figure 6e, Table S1). Residue E237 from IL3 and R333 from H8 of β2AR are involved in salt bridges to residues R375 from the α5-helix and E378 from the C-terminus of Gα_s_, respectively (Figure 6c, Table S1).

For the two D2R-Gα_i_-simulation systems, amino acids important for receptor-G-protein-binding were found to be, in general, qualitatively comparable between D2^Down^R-Gα_i_ and D2^Up^R-Gα_i_. The main difference is caused by the movement of Gα_i_ within the D2^Up^R-Gα_i_-complex discussed above, which weakens the interactions between residues from the extreme N-terminal IL3 and the area around α4-β6. Taken together, residues R132, V136, M140, Y142, R145, R150, R219, R222, K226^Down^, R227^Down^, K367 and K370 from D2R and residues E25, E28, E308^Down^, D315, E318, D341, I344, L348, D350 and L353 from Gα_i_ were revealed to be important for the stability of the complexes. The most interesting observation within the interface of D2R and Gα_i_ is the density of positively charged amino acids from the receptor and of negatively charged amino acids from the G-protein, which mainly form salt bridges to each other. Salt bridges involve residues from IL2/TM4 (R145, R150) and TM5/IL3 (R219, R222, K226^Down^, R227^Down^) of D2R, which are connected to residues from αN-β1 (E25, E28) and α4-β6 (E308^Down^, D315^Down^, E318^Down^)/α5 (D341) of Gα_i_, respectively (Figure 6b, g). The importance of basic amino acids of D2R, which interact with negatively charged residues from Gα_i_, is emphasized by the observation that the alanine scanning mutagenesis for basic amino acids from Gα_i_ (R24, R32, K192, K345, K349) finds a destabilizing effect on the receptor-G-protein-interface. Our results indicate that basic residues from TM6 (K367, K370), despite not forming contacts to acidic amino acids from Gα_i_ (Table S2, S3), participate in stabilizing the receptor-G-protein interface. This can be attributed to interactions with C-terminal residues of Gα_i_, especially F354, where a cation-π-interaction can be formed. As *MMPBSA.py* does not allow alanine scans for terminal residues, it was not possible to perform an alanine scan for this C-terminal residue, but as the corresponding amino acid to F354 is a leucine in Gα_s_ and the amount of direct interactions to surrounding amino acids suggest a great importance for this residue, cation-π-interactions seem to constitute an additional determinant of coupling selectivity. Comparable to residue F139 from β2AR, M140 of D2R is stabilized by a hydrophobic pocket comprised of different amino acids within the two D2R-Gα_i_-simulations (K192, L194, F336 and T340 in D2^Down^R-Gα_i_ and R32, V34, L193 and I343 in D2^Up^R-Gα_i_, Figure 6g, h). These differences are likely to be caused by the movement of Gα_i_ within the D2^Up^R-Gα_i_-simulation (Figure S6). A significant difference between the D2^Down^R-Gα_i_ and D2^Up^R-Gα_i_-complexes lies in the conformation of residue R132 from TM3 (Figure 6a, d). Whereas the side chain of R132 points “downwards” in the direction of the C-terminal α5-helix of Gα_i_ in the D2^Up^R-Gα_i_-complex, its side chain reaches out directly towards the junction of TM7/H8 in the D2^Down^R-Gα_i_-complex. R132 forms a salt bridge to residue D350 from the C-terminus of Gα_i_ in D2^Up^R-Gα_i_. In contrast, R132 and D350 do not show direct D2^Down^R-Gα_i_-interactions. Thus, the conformation of R132 is stabilized by residue F429 from H8 of D2R and D350 of Gα_i_ forms a hydrogen bond to residue N430 of D2R.

### Selectivity Determinants

**Figure 7:**
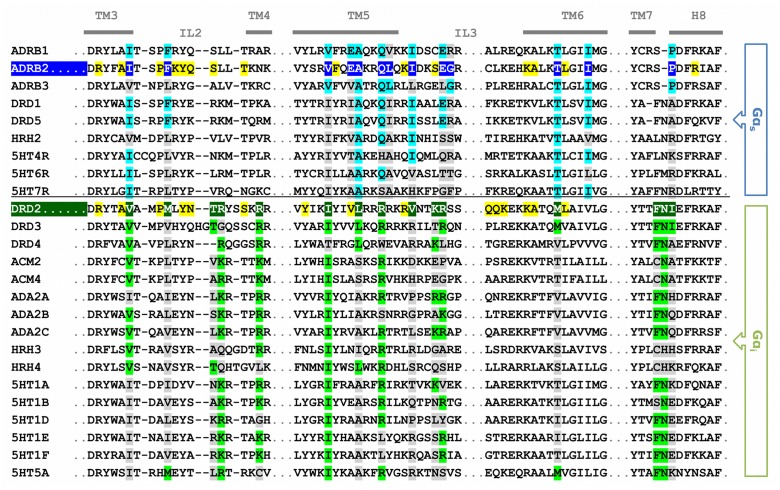
Alignment of contacts areas to G-proteins of aminergic GPCRs. Amino acids of the receptors supposed to determine selective coupling between β2AR-Gα_s_ and D2R-Gα_i_ are highlighted in dark-blue and dark-green, respectively. A brighter color, light-blue or light-green, is attributed to amino acids, which show an identical sequence compared to β2AR and D2R, or, in the case of arginine and lysine residues, a similar sequence, whereas a grey color points to sequence differences. Amino acids, which appear in the interface of β2AR-Gα_s_ and D2R-Gα_i_, but are not supposed to determine selective coupling, are colored in yellow.

Selectivity of a GPCR for a distinct G-protein (or vice versa) arises from structural differences at the interacting epitopes. Figure 5 provides a direct comparison between residues of the β2AR-Gα_s_- and the D2R-Gα_i_-complexes that participate in stabilizing the receptor-G-protein interfaces while showing sequence differences at the same time. Highlighted amino acids of β2AR and D2R are suggested to be crucial for coupling to Gα_s_- and Gα_i_-proteins, respectively, as they exhibit a high degree of sequence conservation within the subfamily of aminergic Gα_s_ and Gα_i_ coupled receptors, which is depicted in Figure 7.

**Figure 8:**
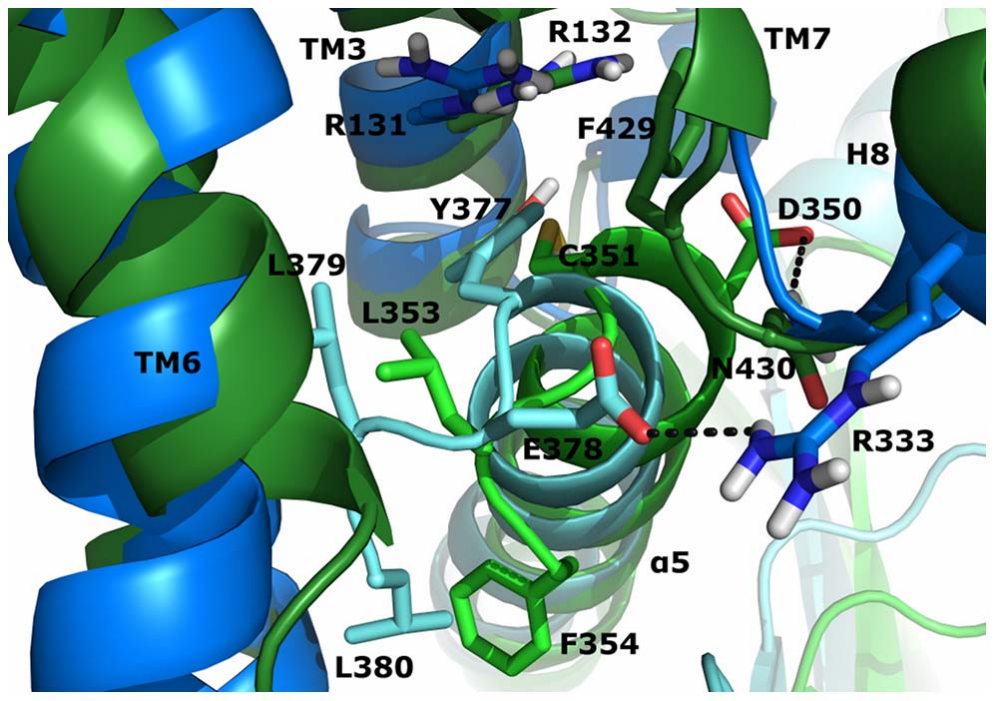
Comparison of the C-terminal parts of Gα_s_ and Gα_i_. The different conformations of the C-termini of Gα_s_ (light-blue ribbons) and Gα_i_ (light-green ribbons) within their pockets in β2AR (dark-blue ribbons) and D2^Down^R (dark-green ribbons), respectively, are shown. Important residues are represented as sticks.

Significant amino acids that control selective receptor-G-protein coupling are located mainly at the intracellular end of TM5 and the N-terminal region of IL3, which comprise a coupling domain for the C-terminal part of Gα and the α4/β6 domain (Figure 5). Interactions of β2AR with the C-terminus of Gα_s_ are supported by residues from TM3-IL2, TM6 and TM7-H8. Among these residues, I135, A226, Q229, I233, E237, T274 and I278 represent a strongly conserved feature of aminergic GPCRs that couple preferentially to Gα_s_ (Figure 7). The equivalent of A226 in TM5 is represented by an alanine residue for every Gα_s_-coupling amine receptor, but differs within the Gα_i_- coupled subfamily. The C-terminal parts of Gα differ significantly (Figure 4, 6). Residues Y377, E378 and L380 as well as D350, C351, G352 and F354 in Gα_s_ and Gα_i_, respectively, are differently stabilized within their GPCR-pockets and lead to a different orientation of their C-termini (Figure 8). Together with residues from the lower parts of the α5-helix (Q370, R371, H373 and R375 in Gα_s_ and I344 and N347 in Gα_i_), which interact with amino acids from the N-terminal part of IL3 of the receptors, they constitute, in general, the main determinant of coupling selectivity of G-proteins. The importance of these regions is supported by mutational studies [37]–[40]. In agreement with functional experiments with artificial model proteins indicating the importance of the N-terminal part of IL3 for D2R coupling [41], [42], the selectivity-determining areas of the D2R-Gα_i_-complexes were found to be located in the intracellular TM5/N-terminal IL3-region of D2R and the C-terminal part of Gα_i_. Selective coupling is supported by the junction of TM3 and IL2, the C-terminal TM6 and the junction of TM7 and helix 8 (Figure 5) when the major amino acids of GPCRs that couple mainly to Gα_i_ were shown to be a valine residue (V136 in D2R) in TM3 and two residues from TM7/H8, F429 and N430 in D2R (Figure 7).

The most striking difference between the β2AR-Gα_s_-complex and the D2R-Gα_i_-complexes was identified for the interaction of the GPCRs’ intracellular loop 2 and the domains αN/β1 and α4/β6 of the G-proteins. Thus, the intracellular loop 2 of β2AR presents a phenylalanine (F139) interacting with a hydrophobic pocket formed by residues from αN-β1, β2-β3 and α5 of Gα_s_ (Figure 6f). Especially the aromatic amino acids H41 and F205 from Gα_s_ are suggested to enable a highly efficient stabilization of the aromatic residue F139 or, to a lesser extent, other bulky, hydrophobic residues in the equivalent position of IL2 in other GPCRs (Figure 5, 7). In contrast to the hydrophobic interaction of β2AR and Gα_s_, D2R and Gα_i_ form ionic interactions between basic amino acids of D2R and negatively charged amino acids of Gα_i_. Ionic contacts involve arginine residues of IL2/TM3 (R145, R150) and TM5/IL3 (R219, K226, R227) of D2R and glutamate residues of αN-β1 (E25, E28) and α4-β6 (E308, E318) of Gα_i_. The importance of these basic amino acids in D2R is proposed to be a general determinant of coupling selectivity towards G_i_, as the structures of G_i_ preferring aminergic GPCRs exhibit homologous residues in the corresponding positions (Figure 7).

### Conclusions

To evaluate receptor binding and activation of unexplored GPCR subtypes and to understand the variety of functionally relevant conformations better, the recent crystal structure of the ternary β2AR-Gα_s_-complex must be complemented by dynamic techniques such as molecular-dynamics simulations, NMR or mass spectroscopy. Because many GPCRs are able to bind more than one G-protein-subtype, models of individual receptor-G-protein complexes will facilitate the rational design of functionally selective drugs inducing the activation of a particular G-protein to a higher extend than coupling to alternative G-protein subtypes. Activation of multiple G-protein dependent and independent pathways and the existence of functionally biased ligands have been demonstrated for the pharmacologically relevant D2R [43]–[45]. The different coupling characteristics of the Gα-subunits Gα_i_ and Gα_o_ towards D2R are associated with subtle sequence differences within their GPCR binding interfaces that involve ionic interactions in Gα_i_ (E28, D315 and D350) missing in Gα_o_ (Figure S9).

Examination of functionally biased ligands in previous studies attributed a major significance to His393^6.55^ in TM6 [21], [23], whose distinct rotamer conformations were herein shown to stabilize different conformations of the ligand-binding pocket of D2R (Figure 3). Thus, His393^6.55^ can act as a switch that connects the behavior of ligands to distinct conformational ensembles on the intracellular side of the receptor. Further MD-simulations with selected ligands and/or G-proteins are therefore necessary to elucidate the impact of His393^6.55^ on functionally selective signaling on a molecular level.

We exploited the crystal structure of the ternary β2AR-Gα_s_-complex to establish an active-state model of the pharmacologically highly relevant dopaminergic D_2_ receptor in complex with the G-protein subunit Gα_i_ and the endogenous ligand dopamine. Different computational methods including molecular-dynamics simulations and computational alanine-scanning mutagenesis were used to identify distinct hot-spot residues that determine receptor-G-protein selectivity (Figure 4, 6). Additionally, we transferred our results to closely related aminergic GPCRs and found highly conserved amino acids of receptor subtypes preferentially coupling to Gα_s_ or Gα_i_ (Figure 7). As structural information for most GPCR-G-protein complexes is still missing, the computational approach described here is of general importance for investigating protein-protein interfaces of ternary complexes and understanding the determinants of functionally selective signaling.

Our computational approach provides firm predictions with respect to amino acids determining selectivity between GPCRs and G-proteins that can now be confirmed experimentally. The impact of water molecules and possible entropic contributions to selective receptor-G-protein coupling were neglected. In the near future, increasing computational power may give the modeling community the opportunity to visualize the activation of a GPCR and its binding to a G-protein in “real time” and to perform such investigations on a higher level of accuracy. A detailed knowledge of the distinct conformational steps involved in receptor activation upon ligand binding and receptor-G-protein coupling will be a prerequisite on the way to fully reveal the secrets of GPCR-signaling.

## Materials and Methods

### Homology Modeling

We used the crystal structure of the β_2_-adrenergic receptor (β2AR) together with a heterotrimeric G-protein [16] (PDB-ID: 3SN6) as a starting point for our calculations. The coordinates of the β2AR and the GαRAS-part of the Gα_s_-protein were used as a template to create a homology model of the dopaminergic D_2_ receptor (D2R) in complex with a Gα_i1_-protein. We omitted the βγ-subunit because it has been shown that the (acylated) α-subunit is sufficient to interact with a G-protein coupled receptor [46]. Three amino acids in the extracellular loop 2 (EL2) of β2AR that are not resolved in the crystal structure were taken from a nanobody-stabilized active-state structure of the β2AR [15] (PDB-ID: 3P0G), the 16 residues missing in the area around α4 of Gα_s_RAS were modeled manually according to the structure of the GTPγS-bound Gα_s_-protein [47] (PDB-ID: 1AZT). The amino-acid sequences for GPCRs and G-proteins were retrieved from the SWISS-PROT database [48]. β2AR and D2R sequences (together with 16 additional sequences of family A GPCRs) as well as Gα_s_ and Gα_i1_ sequences (together with 4 additional Gα protein sequences) were aligned using ClustalX [49] (Gonnet series matrix with a gap open penalty of 10 and a gap extension penalty of 0.2). The initial sequence alignment was manually refined where necessary by means of BioEdit [50] in order to achieve a perfect alignment of the highly conserved amino acids. Absent parts of the β2AR-Gα_s_-complex structure (i.e. intracellular loop 3 of β2AR and Gα_s_AH of Gα_s_) were omitted in the alignment. It has been shown experimentally that removing the bulk of IL3 within the β2AR does not prevent the receptor from coupling to its G-protein [51]. In addition, constructs of the muscarinic receptors M2 and M3, in which the central region of IL3 (more than 100 amino acids) was omitted, were still able to bind their G-proteins selectively and with near wild type efficacy [30], [38]. Therefore, we assume that the truncated D2R used in our investigations is still able to bind to the G_i_-protein selectively, especially as the important N- and C-terminal portions of IL3 are present.

Based on the final alignment and the β2AR-Gα_s_RAS-complex structure as a template, we created 50 models of the D2R-Gα_i_RAS-complex using MODELLER 9v4 [52]. We observed two different rotamer conformations of residue His393^6.55^ in the D2R models with the side chain of His393^6.55^ pointing to the extracellular and intracellular part of the receptor, respectively. We selected two models of the D2R-Gα_i_RAS-complex (referred to as D2^Up^R and D2^Down^R) for further investigation. The models showed the canonical disulfide bond between residue Cys107^3.25^ of transmembrane helix 3 (TM3) and residue Cys182 of extracellular loop 2 (EL2). A second disulfide bond between residues Cys399 and Cys401 of EL3 was attributed to the models because of the spatial proximity of the cysteine residues involved and the observation that the highly homologous dopaminergic D_3_ receptor exhibits a second disulfide bond in an equivalent position [53].

### Structure Refinement and Modification

The two D2R-Gα_i_RAS-complexes were submitted to energy minimization in order to remove bad van der Waals contacts of the amino-acid side chains. The SANDER classic module of the AMBER10 program package was used [54]. We applied 500 steps of steepest descent minimization, followed by 4,500 steps of conjugate gradient minimization. The minimization steps were carried out in a water box with periodic boundary conditions and a nonbonded cutoff of 10.0 Å. The all-atom force field ff99SB [55] was used.

In order to avoid unnecessarily high flexibility during the simulation process caused by open ends in the Gα part of the complexes, we completed the structure of Gα by modeling the missing helical part of Gα_i_ (Gα_i_AH) manually according to the crystal structure of a GDP-bound heterotrimeric Gα_i1_β_1_γ_2_ protein [56] (PDB-ID: 1GP2) and submitted both complexes to energy minimization (see procedure described above). Dopamine was manually docked into D2^Up^R-Gα_i_ and D2^Down^R-Gα_i_RAS to obtain agonist-bound ternary GPCR-G-protein systems. The two nucleotide-free ternary D2R-complexes were minimized with SANDER according to the procedure described above using the general AMBER force field (GAFF) [57] for the dopamine atoms and ff99SB for protein residues. Parameters for dopamine were assigned using antechamber [54] and charges were calculated using Gaussian 09 [58] at the HF/6-31(d,p) level and the RESP procedure according to the literature [59]. A formal charge of +1 was defined for dopamine.

The structural information for the majority of the missing Gα_s_AH in the β2AR-Gα_s_RAS-complex was taken from the crystal structure of the GTPγS-bound Gα_s_-protein (PDB-ID: 1AZT). A small loop of Gα_s_ that connects the Gα_s_RAS- and the Gα_s_AH-subunits, the α1/αA-loop, still not resolved, was modeled manually according to the crystal structure of 1GP2 (residues I55 to K70). Non-conserved residues between Gα_s_ and Gα_i_ were mutated by means of PyMOL [60]. The final structure, comprised of the agonist BI167107, β2AR and the nucleotide-free Gα_s_, was submitted to energy minimization using the procedure described above for the D2R-Gα_i_RAS-systems. Parameters and charges for the ligand BI167107 were used as described above and a formal charge of +1 was attributed to BI167107.

### Preparation of the Simulation Systems

Parameter topology and coordinate files for the minimized complexes (BI167107-β2AR-Gα_s_, dopamine-D2^Up^R-Gα_i_ and dopamine-D2^Down^R-Gα_i_) were build up using the tleap module of AMBER10 and subsequently converted into GROMACS input files [61], [62].

Each complex was inserted into a dioleoylphosphatidylcholine (DOPC) membrane according to a procedure applied successfully earlier [9].

A pre-equilibrated system bearing a hydrated membrane with 72 DOPC lipids [63] was used. This system had to be enlarged in the x, y and z dimensions in order to surround the ternary complexes fully using a method described earlier [9]. The resulting membrane contained 460 DOPC lipids. According to the density profiles of the membrane, the distribution of all components was confirmed to be as expected without water invading the lipophilic parts of the membrane (Figure S3).

The charges of the simulation systems were neutralized by adding 3 sodium and 8 chloride atoms to the β2AR and the D2R complexes, respectively. In total, the BI167107-β2AR-Gα_s_ system consisted of 223,264 atoms (659 amino acids, 49,661 water molecules), the dopamine-D2^Up^R-Gα_i_ system of 227,641 atoms (624 amino acids, 51,333 water molecules) and the dopamine-D2^Down^R-Gα_i_ system of 224,760 atoms (624 amino acids, 50,188 water molecules).

### Membrane Simulations

For all simulations, GAFF was used for the ligands and the DOPC molecules and the force field ff99SB for the protein residues. The SPC/E water model [64] was applied.

After insertion into the prepared membrane, the simulation systems were submitted to energy minimization, equilibration (100 ns) and production molecular-dynamics simulation runs (1 µs) at 310 K using the GROMACS simulation package [61], [62] as described earlier [9]. Initial gaps between GPCRs and DOPC-lipids were shown close perfectly during the equilibration (Figure S2).

Throughout the productive simulations a force of 1.0 kcal mol^−1^ Å^−2^ was applied to the N-terminal part of the G-protein’s αN-helix. In vivo, the αN-helix is anchored to the membrane via acylation with fatty acids and further stabilized by the βγ-subunit when the G-protein is nucleotide-free or bound to GDP [46], [65]. The aim of the applied force is to avoid spurious conformations caused by the high flexibility of the αN-helix in the absence of both the βγ-subunit and the stabilizing acylations because the amino acids that could potentially be acylated are not resolved in the crystal structure of the ternary complex (PDB-ID: 3SN6).

### Data Analysis

The analysis of the trajectories was performed with the PTRAJ module of AMBER10. Calculation of the binding free energies and computational alanine scanning mutagenesis was accomplished using the script MMPBSA.py [35]. As our simulations systems are very large, water molecules had to be deleted from the trajectories before analyzing the data in order to reduce the computational demand of the calculations. Therefore, we cannot preclude the existence of further interactions between GPCRs and G-proteins mediated by water molecules. At least for the interactions revealed by our contact analysis, the interacting amino acids are close enough to each other to form stable interactions, even without water molecules.

Figures were prepared using PyMOL [60].

## Supporting Information

Figure S1 **Conformational changes in the active-state models of the D2R-Gα_i_-complex.** The backbone atoms of GPCRs and G-proteins are shown as ribbons, whereas residue R380 and the nucleotides of the G-proteins are represented as sticks and spheres, respectively. Red arrows denote major helical movements upon receptor activation. (A) Intracellular view of the superposition of active-state models of D2^Down^R (green) and D2^Up^R (dark-red) and the crystal structures of D3R (PDB-ID 3PBL, grey) and β2AR in complex with different binding partners (violet: carazolol, PDB-ID 2RH1; dark-blue: FAUC50, PDB-ID 3PDS; blue: BI167107 and the G_s_ protein, PDB-ID 3SN6). (B) Side view of one part of the receptor-G-protein interface of D2^Down^R-Gα_i_ (green), D2^Up^R-Gα_i_ (dark-red) and β2AR-Gα_s_ (blue). The crystal structures of Gα_i_ in complex with GDP (PDB-ID 1GP2, orange) and of Gα_s_ together with GTPγS (PDB-ID 1AZT, yellow) are aligned on the G-proteins components of the ternary complexes.
(TIFF)

Figure S2 **Equilibration of the simulation systems.** (A) The β2AR-system (blue ribbons) is shown from the top after insertion into the DOPC-bilayer (grey sticks), but before equilibration steps were performed. (B) After equilibration, the gaps between the receptor and the membrane appeared to be perfectly closed. (C) A side view on the β2AR-Gα_s_ simulation system is provided. β2AR and Gα_s_ are shown as blue ribbons. The ligand BI167107 is represented as orange spheres, and the DOPC-molecules as grey sticks. Water molecules are removed for clarity.
(TIFF)

Figure S3 **Density profiles of the simulation systems.** The partial density profiles of individual components of the simulation systems are shown for the simulation time steps 0–100 ns (first 100 ns) and 900–1000 ns (last 100 ns).
(TIFF)

Figure S4 **RMS-deviations within the MD simulations.** (A) The RMS-deviations for the individual components of the β2AR-Gα_s_ system are shown. Values for the ligand BI167107, β2AR and Gα_s_ are given in yellow, dark-blue and light-blue, respectively. (B) The RMS-deviations for the individual components of the D2^Down^R-Gα_i_ system are shown. Values for the ligand dopamine, D2^Down^R and Gα_i_ are given in orange, dark-green and light-green, respectively. (C) The RMS-deviations for the individual components of the D2^Up^R-Gα_i_ system are shown. Values for the ligand dopamine, D2^Up^R and Gα_i_ are given in orange, dark-red and light-red, respectively. The ligands and the receptors are fitted on the Cα-atoms of the receptors, whereas the G-proteins are fitted on the Cα-atoms of the G-proteins. Grey rectangles indicate the time periods used for computational alanine-scanning mutagenesis.
(TIFF)

Figure S5 **Atomic fluctuations within the MD simulations.** The atomic fluctuations for the Cα-atoms of the β2AR-Gα_s_-complex (A), the D2^Down^R-Gα_i_-complex (B) and the D2^Up^R-Gα_i_-complex (C) are given in blue, green and red, respectively. The thickness of the lines indicate different fitting procedures (on Cα-atoms): the thick lines for receptors and G-proteins point to a fit on the receptors and the G-proteins, respectively, whereas the thin lines mean that the G-proteins were fitted on the receptor moieties.
(TIFF)

Figure S6 **Conformational changes of Gα_i_ within the D2^Down/Up^R-Gα_i_-simulations.** (A, B) The D2^Down^R- and the D2^Up^R-Gα_i_-complexes are shown as green and red ribbons, respectively. Residues R227 and D315 are represented as sticks. (C) The distance between the atoms CZ of R227 and CG of D315 is depicted throughout the MD simulations (green: D2^Down^R-Gα_i_, red: D2^Up^R-Gα_i_).
(TIFF)

Figure S7 **Distances between receptors and G-proteins within the MD simulations.** (A) The distances between the centers of mass of β2AR and the whole Gα_s_ and β2AR and the C-terminus of Gα are shown in dark-blue and light-blue, respectively. (B) The distances between the centers of mass of D2^Down^R and the whole Gα_i_ and D2^Down^R and the C-terminus of Gα are shown in dark-green and light-green, respectively. (C) The distances between the centers of mass of D2^Up^R and the whole Gα_i_ and D2^Up^R and the C-terminus of Gα are shown in dark-red and light-red, respectively.
(TIFF)

Figure S8 **Free energies of binding for the ternary complexes.** The free energies of binding for the β2AR-Gα_s_ system (A), for the D2^Down^R-Gα_i_ system (B) and for the D2^Up^R-Gα_i_ system (C) are shown. Here, the free energy of binding consists of a molecular mechanics energy term (internal energy of bonds, angles and dihedrals), the polar contribution and the nonpolar contribution of the solvation free energy (polar contribution calculated using the Generalized Born equation and the nonpolar contribution using the molecular solvent-accessible surface area). The curves exhibit a best fit line with a positive gradient for (A) and (B) (0.012 and 0.021 for the β2AR-Gα_s_- and the D2^Down^R-Gα_i_-system, respectively), and a negative gradient for curve (C) (−0.021 for the D2^Up^R-Gα_i_-system). As these gradients are very small, we expect that the values will converge to zero for longer simulation times.
(TIFF)

Figure S9 **Alignment of contact areas of chosen Gα-subunits.** Amino acids within the Gα_s_ and Gα_i_ sequences forming stable contacts to receptor residues are highlighted with a blue and green background, respectively (according to Figure 4). Red backgrounds point to sequence differences between Gα_i_ and Gα_o_ subunits. Red letters indicate residues involved in ionic interactions.
(TIFF)

Table S1 **Amino-acid contacts within the β2AR-Gα_s_-simulation.** The occurrence for each amino-acid contact throughout the MD simulation is shown in the grey columns.
(DOC)

Table S2 **Amino-acid contacts within the D2^Down^R-Gα_i_-simulation.** The occurrence for each amino-acid contact throughout the MD simulation is shown in the grey columns.
(DOC)

Table S3 **Amino-acid contacts within the D2^Up^R-Gα_i_-simulation.** The occurrence for each amino-acid contact throughout the MD simulation is shown in the grey columns.
(DOC)

Table S4 **Results of the computational alanine scanning for the receptors and the G-proteins.** aa refers to the amino acids mutated to alanine. ΔΔG-values are provided in the format ‘value ± standard deviation’. The left column shows the regions within the GPCRs and the G-proteins, to which the mutated amino acids belong.
(DOC)

## References

[1] Fredriksson R, Lagerstrom MC, Lundin LG, Schioth HB (2003) The G-protein-coupled receptors in the human genome form five main families. Phylogenetic analysis, paralogon groups, and fingerprints. Mol Pharmacol 63: 1256–1272. 12761335 10.1124/mol.63.6.1256

[2] Lagerstrom MC, Schioth HB (2008) Structural diversity of G protein-coupled receptors and significance for drug discovery. Nat Rev Drug Discov 7: 339–357. 18382464 10.1038/nrd2518

[3] Ahuja S, Smith SO (2009) Multiple switches in G protein-coupled receptor activation. Trends Pharmacol Sci 30: 494–502. 19732972 10.1016/j.tips.2009.06.003

[4] Nygaard R, Frimurer TM, Holst B, Rosenkilde MM, Schwartz TW (2009) Ligand binding and micro-switches in 7TM receptor structures. Trends Pharmacol Sci 30: 249–259. 19375807 10.1016/j.tips.2009.02.006

[5] Trzaskowski B, Latek D, Yuan S, Ghoshdastider U, Debinski A, et al (2012) Action of molecular switches in GPCRs–theoretical and experimental studies. Curr Med Chem 19: 1090–1109. 22300046 10.2174/092986712799320556 PMC3343417

[6] Dror RO, Arlow DH, Maragakis P, Mildorf TJ, Pan AC, et al (2011) Activation mechanism of the beta2-adrenergic receptor. Proceedings of the National Academy of Sciences of the United States of America 108: 18684–18689. 22031696 10.1073/pnas.1110499108 PMC3219117

[7] Venkatakrishnan AJ, Deupi X, Lebon G, Tate CG, Schertler GF, et al (2013) Molecular signatures of G-protein-coupled receptors. Nature 494: 185–194. 23407534 10.1038/nature11896

[8] Deupi X, Standfuss J, Schertler G (2012) Conserved activation pathways in G-protein-coupled receptors. Biochem Soc Trans 40: 383–388. 22435816 10.1042/BST20120001

[9] Goetz A, Lanig H, Gmeiner P, Clark T (2011) Molecular Dynamics Simulations of the Effect of the G-Protein and Diffusible Ligands on the β2-Adrenergic Receptor. Journal of molecular biology. 10.1016/j.jmb.2011.10.015 22037586

[10] De Lean A, Stadel JM, Lefkowitz RJ (1980) A ternary complex model explains the agonist-specific binding properties of the adenylate cyclase-coupled beta-adrenergic receptor. J Biol Chem 255: 7108–7117. 6248546

[11] Katritch V, Cherezov V, Stevens RC (2012) Structure-Function of the G Protein-Coupled Receptor Superfamily. Annu Rev Pharmacol Toxicol. 10.1146/annurev-pharmtox-032112-135923 PMC3540149 23140243

[12] Rasmussen SG, Choi HJ, Rosenbaum DM, Kobilka TS, Thian FS, et al (2007) Crystal structure of the human beta2 adrenergic G-protein-coupled receptor. Nature 450: 383–387. 17952055 10.1038/nature06325

[13] Cherezov V, Rosenbaum DM, Hanson MA, Rasmussen SG, Thian FS, et al (2007) High-resolution crystal structure of an engineered human beta2-adrenergic G protein-coupled receptor. Science 318: 1258–1265. 17962520 10.1126/science.1150577 PMC2583103

[14] Rosenbaum DM, Zhang C, Lyons JA, Holl R, Aragao D, et al (2011) Structure and function of an irreversible agonist-beta(2) adrenoceptor complex. Nature 469: 236–240. 21228876 10.1038/nature09665 PMC3074335

[15] Rasmussen SG, Choi HJ, Fung JJ, Pardon E, Casarosa P, et al (2011) Structure of a nanobody-stabilized active state of the beta(2) adrenoceptor. Nature 469: 175–180. 21228869 10.1038/nature09648 PMC3058308

[16] Rasmussen SG, DeVree BT, Zou Y, Kruse AC, Chung KY, et al (2011) Crystal structure of the beta2 adrenergic receptor-Gs protein complex. Nature 477: 549–555. 21772288 10.1038/nature10361 PMC3184188

[17] Nygaard R, Zou Y, Dror RO, Mildorf TJ, Arlow DH, et al (2013) The Dynamic Process of beta(2)-Adrenergic Receptor Activation. Cell 152: 532–542. 23374348 10.1016/j.cell.2013.01.008 PMC3586676

[18] Kolb P, Rosenbaum DM, Irwin JJ, Fung JJ, Kobilka BK, et al (2009) Structure-based discovery of beta2-adrenergic receptor ligands. Proc Natl Acad Sci U S A 106: 6843–6848. 19342484 10.1073/pnas.0812657106 PMC2672528

[19] Neve KA (2010) The dopamine receptors. New York, NY: Humana Press. xii, 647 p. p.

[20] Ehrlich K, Gotz A, Bollinger S, Tschammer N, Bettinetti L, et al (2009) Dopamine D2, D3, and D4 selective phenylpiperazines as molecular probes to explore the origins of subtype specific receptor binding. J Med Chem 52: 4923–4935. 19606869 10.1021/jm900690y

[21] Tschammer N, Bollinger S, Kenakin T, Gmeiner P (2011) Histidine 6.55 is a major determinant of ligand-biased signaling in dopamine D2L receptor. Mol Pharmacol 79: 575–585. 21163968 10.1124/mol.110.068106

[22] Tschammer N, Elsner J, Goetz A, Ehrlich K, Schuster S, et al (2011) Highly potent 5-aminotetrahydropyrazolopyridines: enantioselective dopamine D3 receptor binding, functional selectivity, and analysis of receptor-ligand interactions. J Med Chem 54: 2477–2491. 21388142 10.1021/jm101639t

[23] Fowler JC, Bhattacharya S, Urban JD, Vaidehi N, Mailman RB (2012) Receptor Conformations Involved in Dopamine D2L Receptor Functional Selectivity Induced by Selected Transmembrane 5 Serine Mutations. Mol Pharmacol. 10.1124/mol.111.075457 PMC3362898 22416052

[24] Coley C, Woodward R, Johansson AM, Strange PG, Naylor LH (2000) Effect of multiple serine/alanine mutations in the transmembrane spanning region V of the D2 dopamine receptor on ligand binding. Journal of neurochemistry 74: 358–366. 10617140 10.1046/j.1471-4159.2000.0740358.x

[25] Warne T, Moukhametzianov R, Baker JG, Nehme R, Edwards PC, et al (2011) The structural basis for agonist and partial agonist action on a beta(1)-adrenergic receptor. Nature 469: 241–244. 21228877 10.1038/nature09746 PMC3023143

[26] Van Eps N, Preininger AM, Alexander N, Kaya AI, Meier S, et al (2011) Interaction of a G protein with an activated receptor opens the interdomain interface in the alpha subunit. Proceedings of the National Academy of Sciences of the United States of America 108: 9420–9424. 21606326 10.1073/pnas.1105810108 PMC3111277

[27] Westfield GH, Rasmussen SG, Su M, Dutta S, Devree BT, et al (2011) Structural flexibility of the G{alpha}s {alpha}-helical domain in the {beta}2-adrenoceptor Gs complex. Proceedings of the National Academy of Sciences of the United States of America 108: 16086–16091. 21914848 10.1073/pnas.1113645108 PMC3179071

[28] Konig B, Arendt A, McDowell JH, Kahlert M, Hargrave PA, et al (1989) Three cytoplasmic loops of rhodopsin interact with transducin. Proc Natl Acad Sci U S A 86: 6878–6882. 2780545 10.1073/pnas.86.18.6878 PMC297953

[29] Hamm HE, Deretic D, Arendt A, Hargrave PA, Koenig B, et al (1988) Site of G protein binding to rhodopsin mapped with synthetic peptides from the alpha subunit. Science 241: 832–835. 3136547 10.1126/science.3136547

[30] Hu J, Wang Y, Zhang X, Lloyd JR, Li JH, et al (2010) Structural basis of G protein-coupled receptor-G protein interactions. Nature chemical biology 6: 541–548. 20512139 10.1038/nchembio.385 PMC3104732

[31] Scheerer P, Park JH, Hildebrand PW, Kim YJ, Krauss N, et al (2008) Crystal structure of opsin in its G-protein-interacting conformation. Nature 455: 497–502. 18818650 10.1038/nature07330

[32] Massova I, Kollman PA (1999) Computational Alanine Scanning To Probe Protein−Protein Interactions: A Novel Approach To Evaluate Binding Free Energies. Journal of the American Chemical Society 121: 8133–8143.

[33] Bradshaw RT, Patel BH, Tate EW, Leatherbarrow RJ, Gould IR (2011) Comparing experimental and computational alanine scanning techniques for probing a prototypical protein-protein interaction. Protein Eng Des Sel 24: 197–207. 20656696 10.1093/protein/gzq047

[34] Kollman PA, Massova I, Reyes C, Kuhn B, Huo S, et al (2000) Calculating Structures and Free Energies of Complex Molecules: Combining Molecular Mechanics and Continuum Models. Accounts of Chemical Research 33: 889–897. 11123888 10.1021/ar000033j

[35] Miller Iii BR, McGee TD, Swails JM, Homeyer N, Gohlke H, et al.. (2012) MMPBSA.py: An Efficient Program for End-State Free Energy Calculations. Journal of Chemical Theory and Computation. 10.1021/ct300418h 26605738

[36] Moro O, Lameh J, Hogger P, Sadee W (1993) Hydrophobic amino acid in the i2 loop plays a key role in receptor-G protein coupling. J Biol Chem 268: 22273–22276. 8226735

[37] Conklin BR, Farfel Z, Lustig KD, Julius D, Bourne HR (1993) Substitution of three amino acids switches receptor specificity of Gq alpha to that of Gi alpha. Nature 363: 274–276. 8387644 10.1038/363274a0

[38] Lechleiter J, Hellmiss R, Duerson K, Ennulat D, David N, et al (1990) Distinct sequence elements control the specificity of G protein activation by muscarinic acetylcholine receptor subtypes. EMBO J 9: 4381–4390. 2124972 10.1002/j.1460-2075.1990.tb07888.x PMC552228

[39] Kobilka BK, Kobilka TS, Daniel K, Regan JW, Caron MG, et al (1988) Chimeric alpha 2-,beta 2-adrenergic receptors: delineation of domains involved in effector coupling and ligand binding specificity. Science 240: 1310–1316. 2836950 10.1126/science.2836950

[40] Eason MG, Liggett SB (1995) Identification of a Gs coupling domain in the amino terminus of the third intracellular loop of the alpha 2A-adrenergic receptor. Evidence for distinct structural determinants that confer Gs versus Gi coupling. J Biol Chem 270: 24753–24760. 7559592 10.1074/jbc.270.42.24753

[41] Voss T, Wallner E, Czernilofsky AP, Freissmuth M (1993) Amphipathic alpha-helical structure does not predict the ability of receptor-derived synthetic peptides to interact with guanine nucleotide-binding regulatory proteins. J Biol Chem 268: 4637–4642. 8383121

[42] Nanoff C, Koppensteiner R, Yang Q, Fuerst E, Ahorn H, et al (2006) The carboxyl terminus of the Galpha-subunit is the latch for triggered activation of heterotrimeric G proteins. Molecular pharmacology 69: 397–405. 16210429 10.1124/mol.105.016725

[43] Nickolls SA, Strange PG (2003) Interaction of the D2short dopamine receptor with G proteins: analysis of receptor/G protein selectivity. Biochem Pharmacol 65: 1139–1150. 12663049 10.1016/s0006-2952(03)00040-6

[44] Gazi L, Nickolls SA, Strange PG (2003) Functional coupling of the human dopamine D2 receptor with G alpha i1, G alpha i2, G alpha i3 and G alpha o G proteins: evidence for agonist regulation of G protein selectivity. Br J Pharmacol 138: 775–786. 12642378 10.1038/sj.bjp.0705116 PMC1573727

[45] Lane JR, Powney B, Wise A, Rees S, Milligan G (2007) Protean agonism at the dopamine D2 receptor: (S)-3-(3-hydroxyphenyl)-N-propylpiperidine is an agonist for activation of Go1 but an antagonist/inverse agonist for Gi1,Gi2, and Gi3. Mol Pharmacol 71: 1349–1359. 17287401 10.1124/mol.106.032722

[46] Herrmann R, Heck M, Henklein P, Hofmann KP, Ernst OP (2006) Signal transfer from GPCRs to G proteins: role of the G alpha N-terminal region in rhodopsin-transducin coupling. J Biol Chem 281: 30234–30241. 16847064 10.1074/jbc.M600797200

[47] Sunahara RK, Tesmer JJ, Gilman AG, Sprang SR (1997) Crystal structure of the adenylyl cyclase activator Gsalpha. Science 278: 1943–1947. 9395396 10.1126/science.278.5345.1943

[48] Bairoch A, Apweiler R (2000) The SWISS-PROT protein sequence database and its supplement TrEMBL in 2000. Nucleic Acids Res 28: 45–48. 10592178 10.1093/nar/28.1.45 PMC102476

[49] Larkin MA, Blackshields G, Brown NP, Chenna R, McGettigan PA, et al (2007) Clustal W and Clustal X version 2.0. Bioinformatics 23: 2947–2948. 17846036 10.1093/bioinformatics/btm404

[50] Hall TA (1999) BioEdit: a user-friendly biological sequence alignment editor and analysis program for Windows 95/98/NT. Nucleic Acids Symposium Series 41: 95–98.

[51] Rubenstein RC, Wong SK, Ross EM (1987) The hydrophobic tryptic core of the beta-adrenergic receptor retains Gs regulatory activity in response to agonists and thiols. J Biol Chem 262: 16655–16662. 2890639

[52] Sali A, Blundell TL (1993) Comparative protein modelling by satisfaction of spatial restraints. J Mol Biol 234: 779–815. 8254673 10.1006/jmbi.1993.1626

[53] Chien EY, Liu W, Zhao Q, Katritch V, Han GW, et al (2010) Structure of the human dopamine D3 receptor in complex with a D2/D3 selective antagonist. Science 330: 1091–1095. 21097933 10.1126/science.1197410 PMC3058422

[54] D.A. Case TAD, T.E Cheatham, III, C.L Simmerling, J Wang, R.E Duke, R Luo, M Crowley, R.C Walker, W Zhang, K.M Merz, B Wang, S Hayik, A Roitberg, G Seabra, I Kolossváry, K.F Wong, F Paesani, J Vanicek, X Wu, S.R Brozell, T Steinbrecher, H Gohlke, L Yang, C Tan, J Mongan, V Hornak, G Cui, D.H Mathews, M.G Seetin, C Sagui, V Babin, and P.A Kollman (2008) AMBER 10.

[55] Hornak V, Abel R, Okur A, Strockbine B, Roitberg A, et al (2006) Comparison of multiple Amber force fields and development of improved protein backbone parameters. Proteins 65: 712–725. 16981200 10.1002/prot.21123 PMC4805110

[56] Wall MA, Coleman DE, Lee E, Iniguez-Lluhi JA, Posner BA, et al (1995) The structure of the G protein heterotrimer Gi alpha 1 beta 1 gamma 2. Cell 83: 1047–1058. 8521505 10.1016/0092-8674(95)90220-1

[57] Wang J, Wolf RM, Caldwell JW, Kollman PA, Case DA (2004) Development and testing of a general amber force field. J Comput Chem 25: 1157–1174. 15116359 10.1002/jcc.20035

[58] Frisch MJ, Trucks GW, Schlegel HB, Scuseria GE, Robb MA, et al.. (2009) Gaussian 09, Revision B.01. Wallingford CT.

[59] Bayly CI, Cieplak P, Cornell W, Kollman PA (1993) A well-behaved electrostatic potential based method using charge restraints for deriving atomic charges: the RESP model. The Journal of Physical Chemistry 97: 10269–10280.

[60] Schrodinger LLC (2010) The PyMOL Molecular Graphics System, Version 1.3r1.

[61] Hess B, Kutzner C, van der Spoel D, Lindahl E (2008) GROMACS 4: Algorithms for Highly Efficient, Load-Balanced, and Scalable Molecular Simulation. Journal of Chemical Theory and Computation 4: 435–447. 26620784 10.1021/ct700301q

[62] Van Der Spoel D, Lindahl E, Hess B, Groenhof G, Mark AE, et al (2005) GROMACS: fast, flexible, and free. J Comput Chem 26: 1701–1718. 16211538 10.1002/jcc.20291

[63] Siu SW, Vacha R, Jungwirth P, Bockmann RA (2008) Biomolecular simulations of membranes: physical properties from different force fields. J Chem Phys 128: 125103. 18376978 10.1063/1.2897760

[64] Berendsen HJC, Grigera JR, Straatsma TP (1987) The missing term in effective pair potentials. The Journal of Physical Chemistry 91: 6269–6271.

[65] Preininger AM, Van Eps N, Yu N-J, Medkova M, Hubbell WL, et al (2003) The Myristoylated Amino Terminus of Gαi1 Plays a Critical Role in the Structure and Function of Gαi1 Subunits in Solution. Biochemistry 42: 7931–7941. 12834345 10.1021/bi0345438

