# Supplementary figures and images for: Active-State Models of Ternary GPCR Complexes: Determinants of Selective Receptor-G-Protein Coupling
Source: PLoS One. 2013 Jun 24;8(6):e67244. doi: 10.1371/journal.pone.0067244 (PMC3691126; doi:10.1371/journal.pone.0067244)

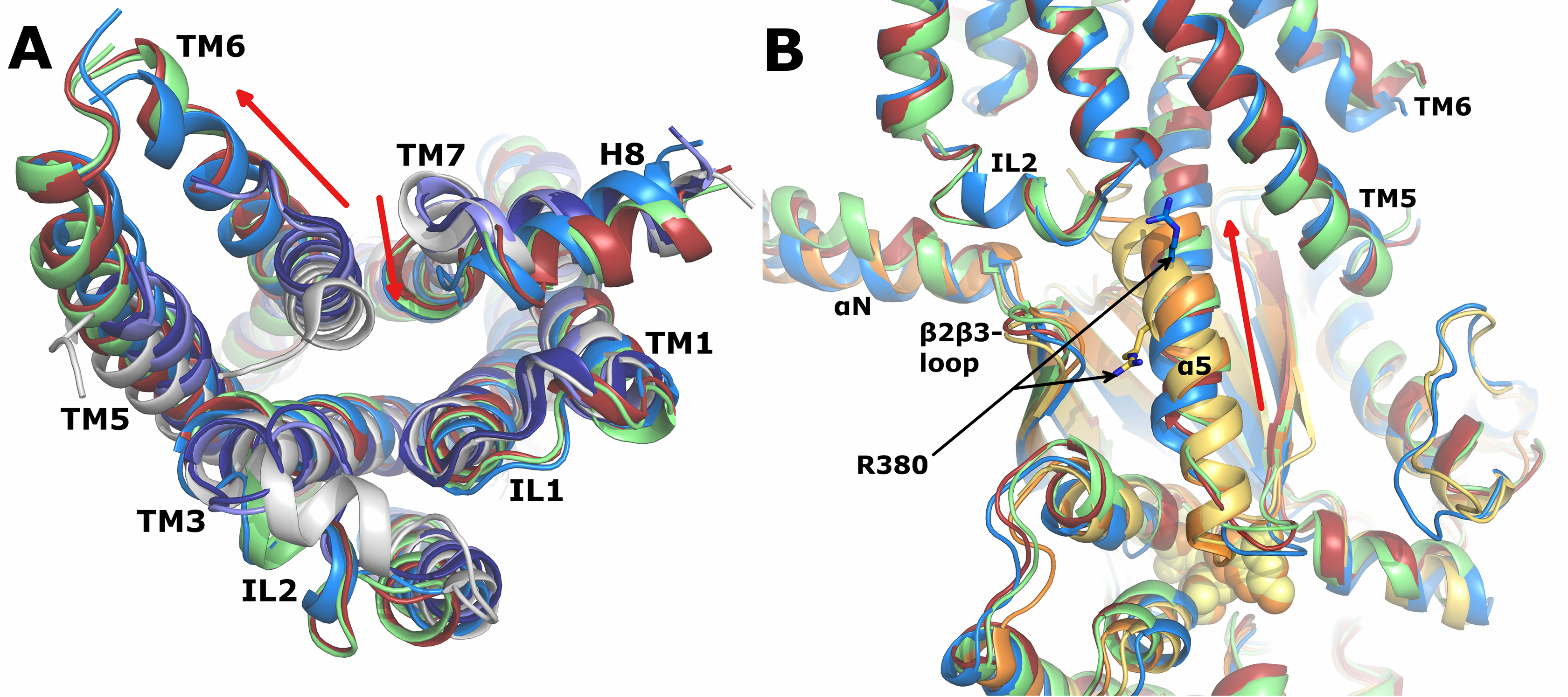

Supplement: Figure S1 — Conformational changes in the active-state models of the D2R-Gαi-complex. The backbone atoms of GPCRs and G-proteins are shown as ribbons, whereas residue R380 and the nucleotides of the G-proteins are represented as sticks and spheres, respectively. Red arrows denote major helical movements upon receptor activation. (A) Intracellular view of the superposition of active-state models of D2DownR (green) and D2UpR (dark-red) and the crystal structures of D3R (PDB-ID 3PBL, grey) and β2AR in complex with different binding partners (violet: carazolol, PDB-ID 2RH1; dark-blue: FAUC50, PDB-ID 3PDS; blue: BI167107 and the Gs protein, PDB-ID 3SN6). (B) Side view of one part of the receptor-G-protein interface of D2DownR-Gαi (green), D2UpR-Gαi (dark-red) and β2AR-Gαs (blue). The crystal structures of Gαi in complex with GDP (PDB-ID 1GP2, orange) and of Gαs together with GTPγS (PDB-ID 1AZT, yellow) are aligned on the G-proteins components of the ternary complexes. (TIFF) [file pone.0067244.s001.tiff]

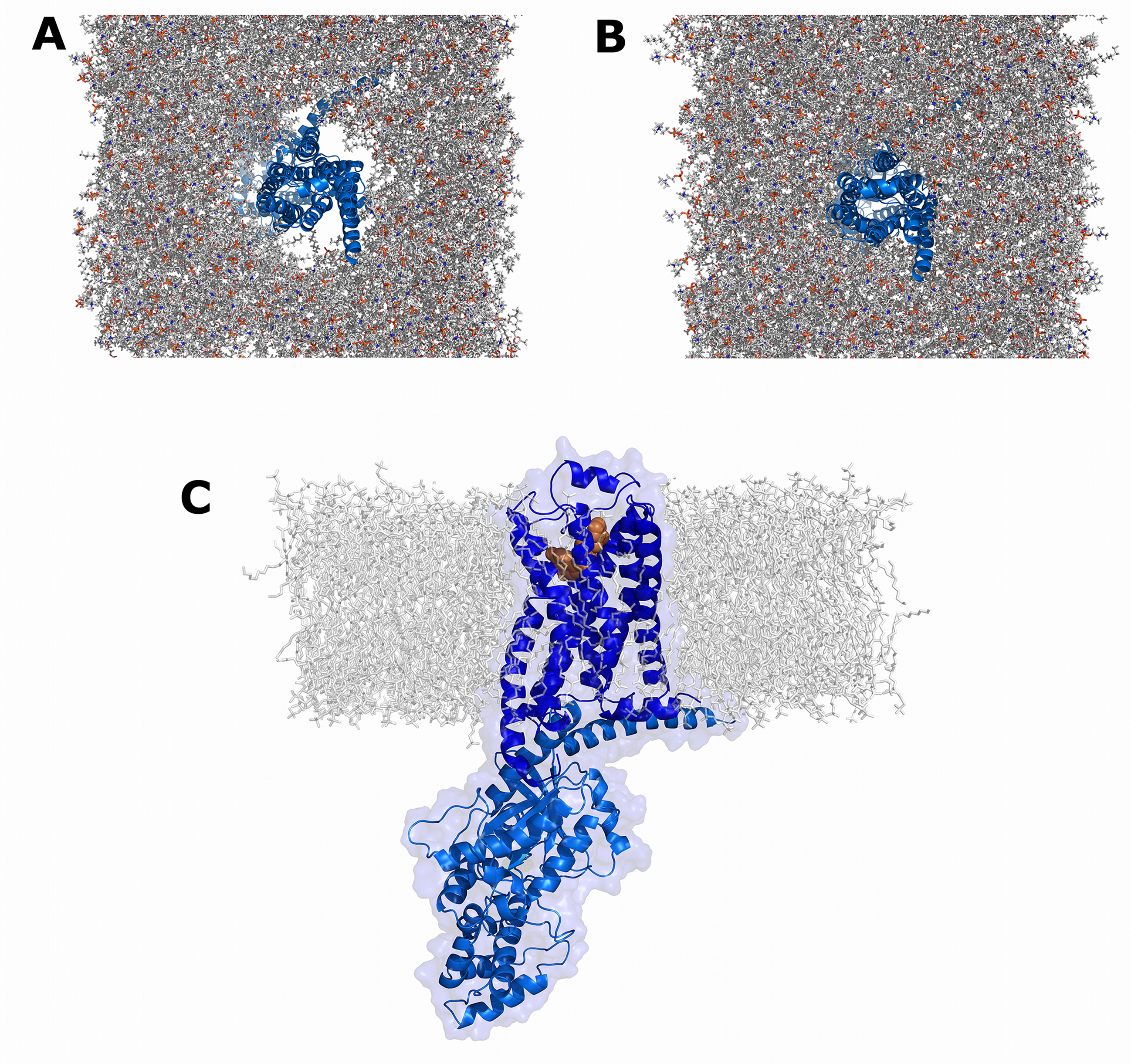

Supplement: Figure S2 — Equilibration of the simulation systems. (A) The β2AR-system (blue ribbons) is shown from the top after insertion into the DOPC-bilayer (grey sticks), but before equilibration steps were performed. (B) After equilibration, the gaps between the receptor and the membrane appeared to be perfectly closed. (C) A side view on the β2AR-Gαs simulation system is provided. β2AR and Gαs are shown as blue ribbons. The ligand BI167107 is represented as orange spheres, and the DOPC-molecules as grey sticks. Water molecules are removed for clarity. (TIFF) [file pone.0067244.s002.tiff]

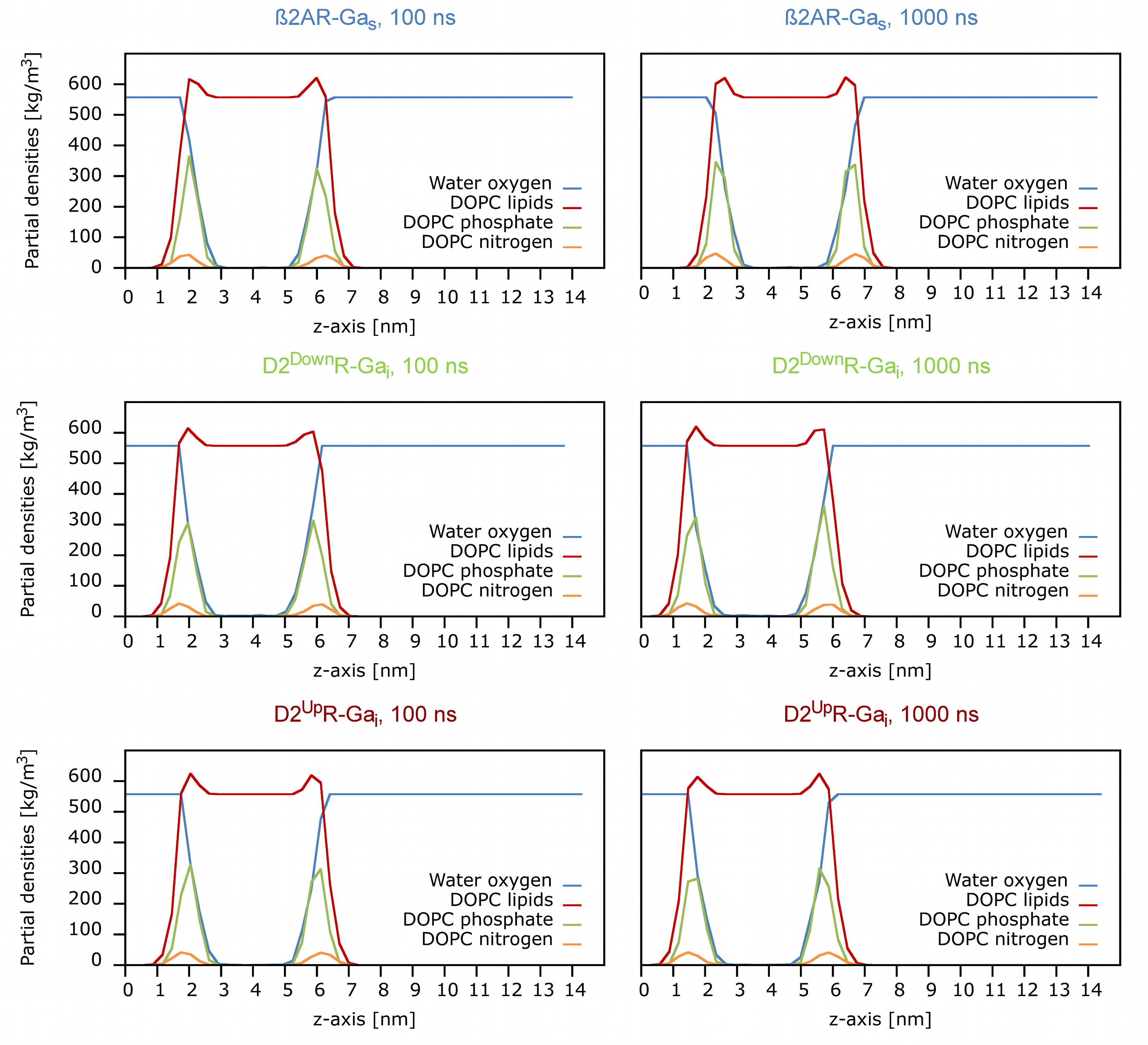

Supplement: Figure S3 — Density profiles of the simulation systems. The partial density profiles of individual components of the simulation systems are shown for the simulation time steps 0–100 ns (first 100 ns) and 900–1000 ns (last 100 ns). (TIFF) [file pone.0067244.s003.tiff]

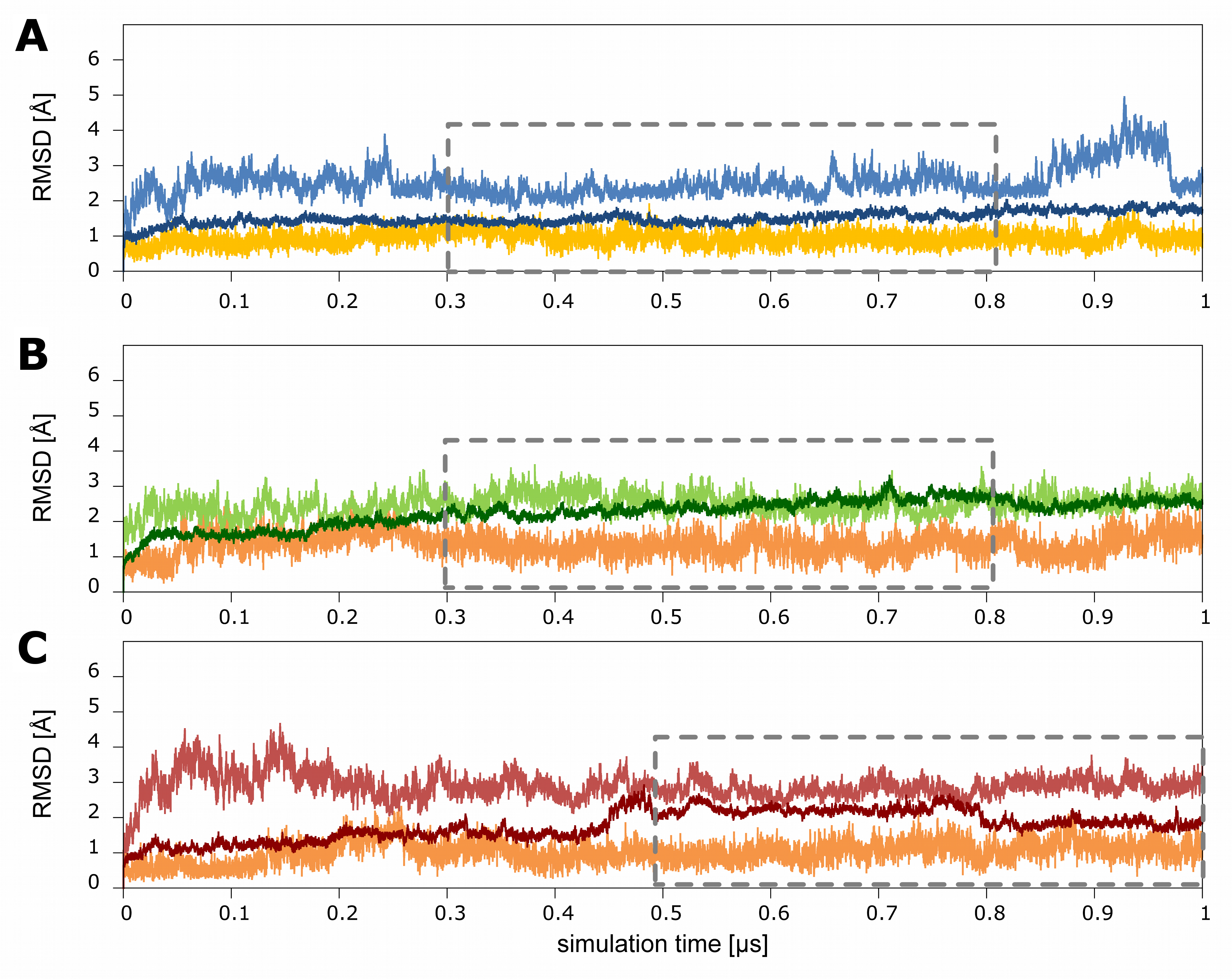

Supplement: Figure S4 — RMS-deviations within the MD simulations. (A) The RMS-deviations for the individual components of the β2AR-Gαs system are shown. Values for the ligand BI167107, β2AR and Gαs are given in yellow, dark-blue and light-blue, respectively. (B) The RMS-deviations for the individual components of the D2DownR-Gαi system are shown. Values for the ligand dopamine, D2DownR and Gαi are given in orange, dark-green and light-green, respectively. (C) The RMS-deviations for the individual components of the D2UpR-Gαi system are shown. Values for the ligand dopamine, D2UpR and Gαi are given in orange, dark-red and light-red, respectively. The ligands and the receptors are fitted on the Cα-atoms of the receptors, whereas the G-proteins are fitted on the Cα-atoms of the G-proteins. Grey rectangles indicate the time periods used for computational alanine-scanning mutagenesis. (TIFF) [file pone.0067244.s004.tiff]

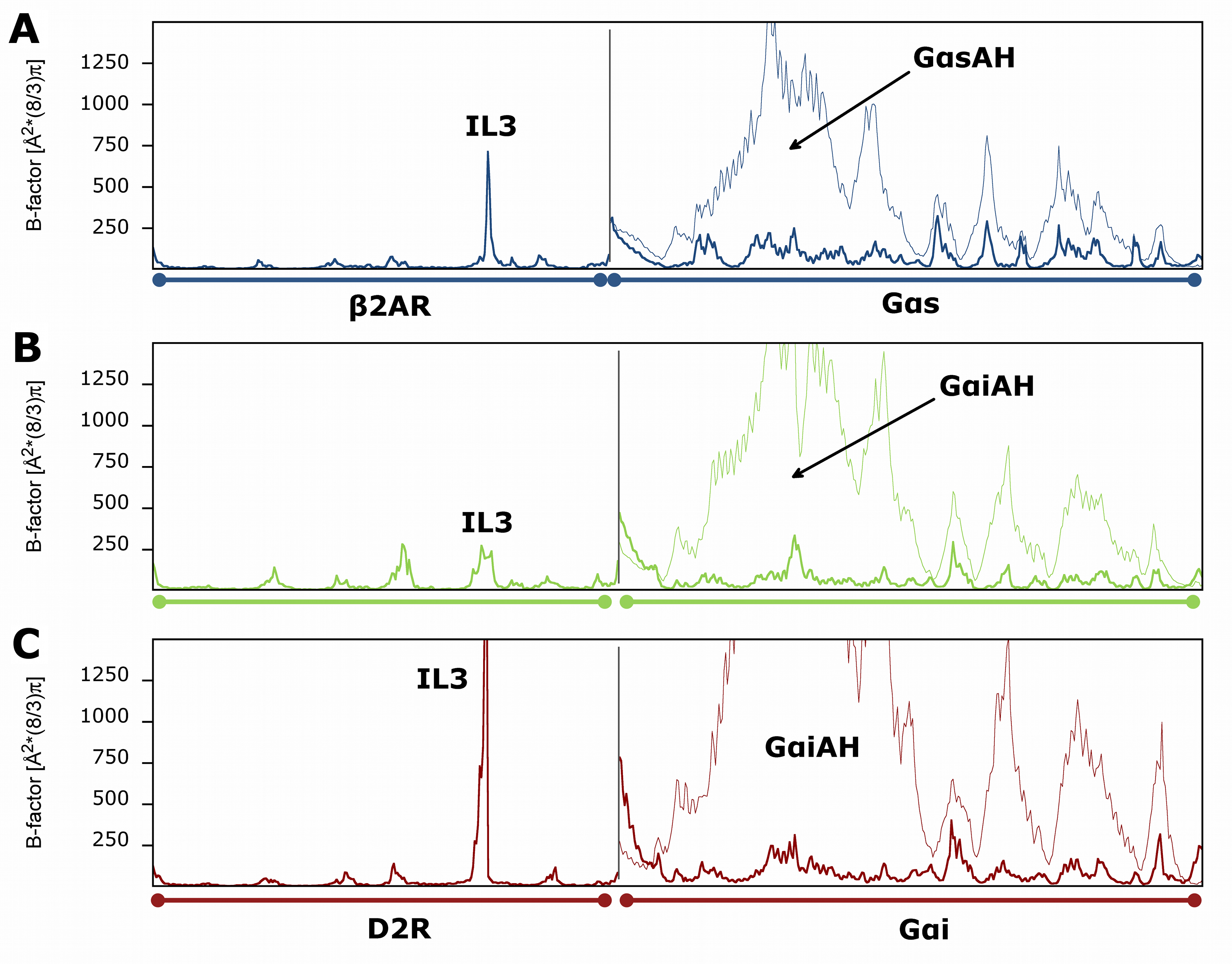

Supplement: Figure S5 — Atomic fluctuations within the MD simulations. The atomic fluctuations for the Cα-atoms of the β2AR-Gαs-complex (A), the D2DownR-Gαi-complex (B) and the D2UpR-Gαi-complex (C) are given in blue, green and red, respectively. The thickness of the lines indicate different fitting procedures (on Cα-atoms): the thick lines for receptors and G-proteins point to a fit on the receptors and the G-proteins, respectively, whereas the thin lines mean that the G-proteins were fitted on the receptor moieties. (TIFF) [file pone.0067244.s005.tiff]

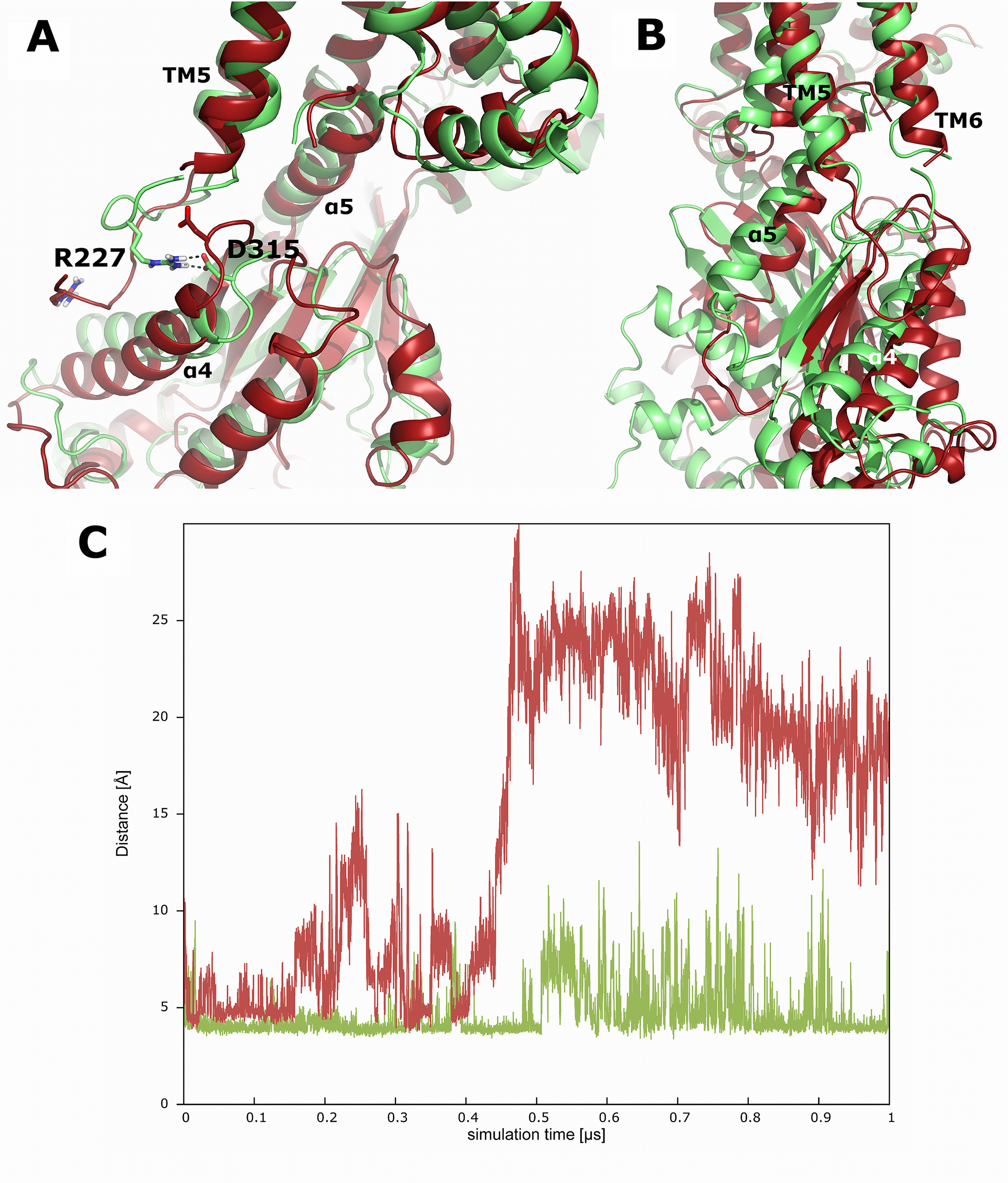

Supplement: Figure S6 — Conformational changes of Gαi within the D2Down/UpR-Gαi-simulations. (A, B) The D2DownR- and the D2UpR-Gαi-complexes are shown as green and red ribbons, respectively. Residues R227 and D315 are represented as sticks. (C) The distance between the atoms CZ of R227 and CG of D315 is depicted throughout the MD simulations (green: D2DownR-Gαi, red: D2UpR-Gαi). (TIFF) [file pone.0067244.s006.tiff]

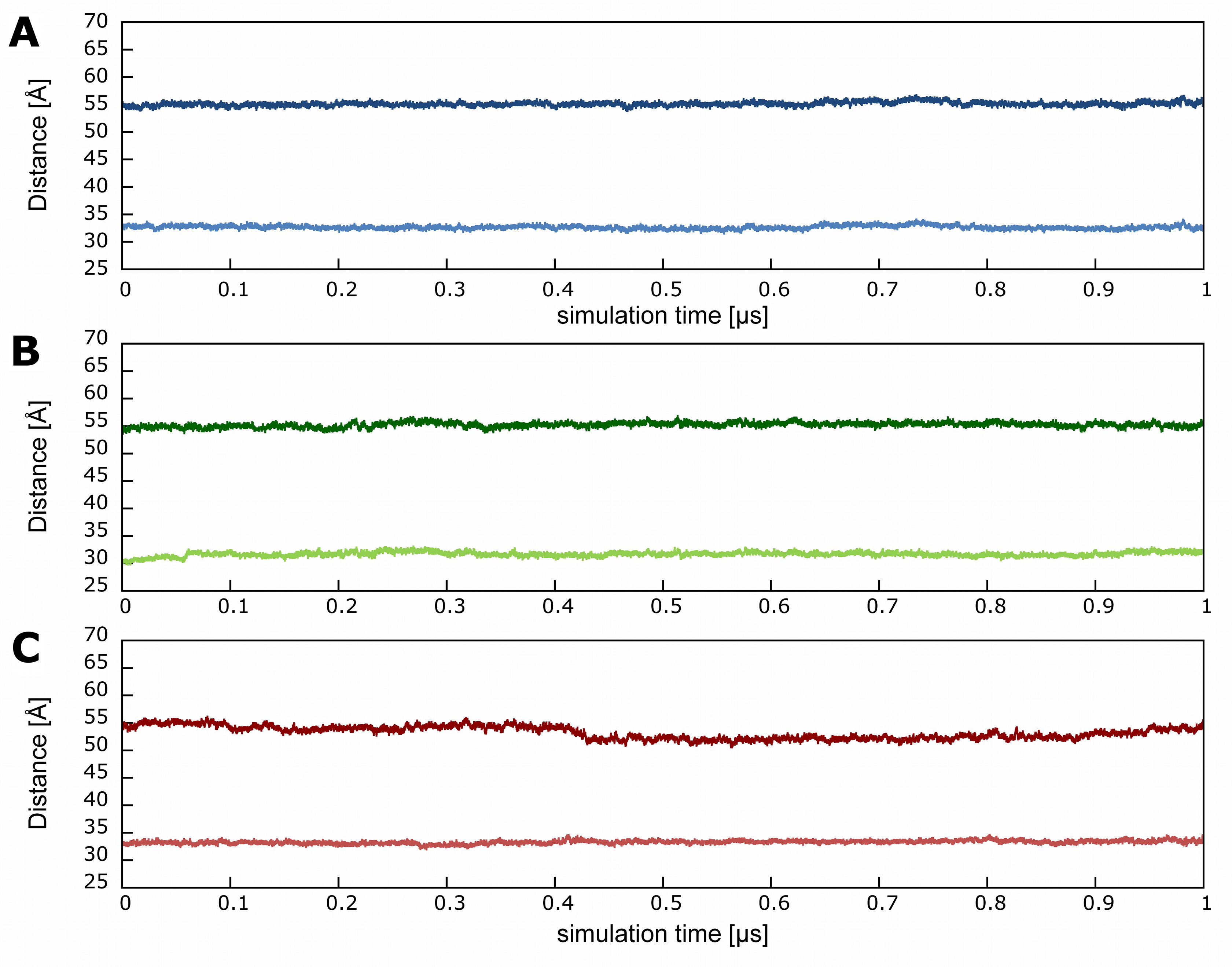

Supplement: Figure S7 — Distances between receptors and G-proteins within the MD simulations. (A) The distances between the centers of mass of β2AR and the whole Gαs and β2AR and the C-terminus of Gα are shown in dark-blue and light-blue, respectively. (B) The distances between the centers of mass of D2DownR and the whole Gαi and D2DownR and the C-terminus of Gα are shown in dark-green and light-green, respectively. (C) The distances between the centers of mass of D2UpR and the whole Gαi and D2UpR and the C-terminus of Gα are shown in dark-red and light-red, respectively. (TIFF) [file pone.0067244.s007.tiff]

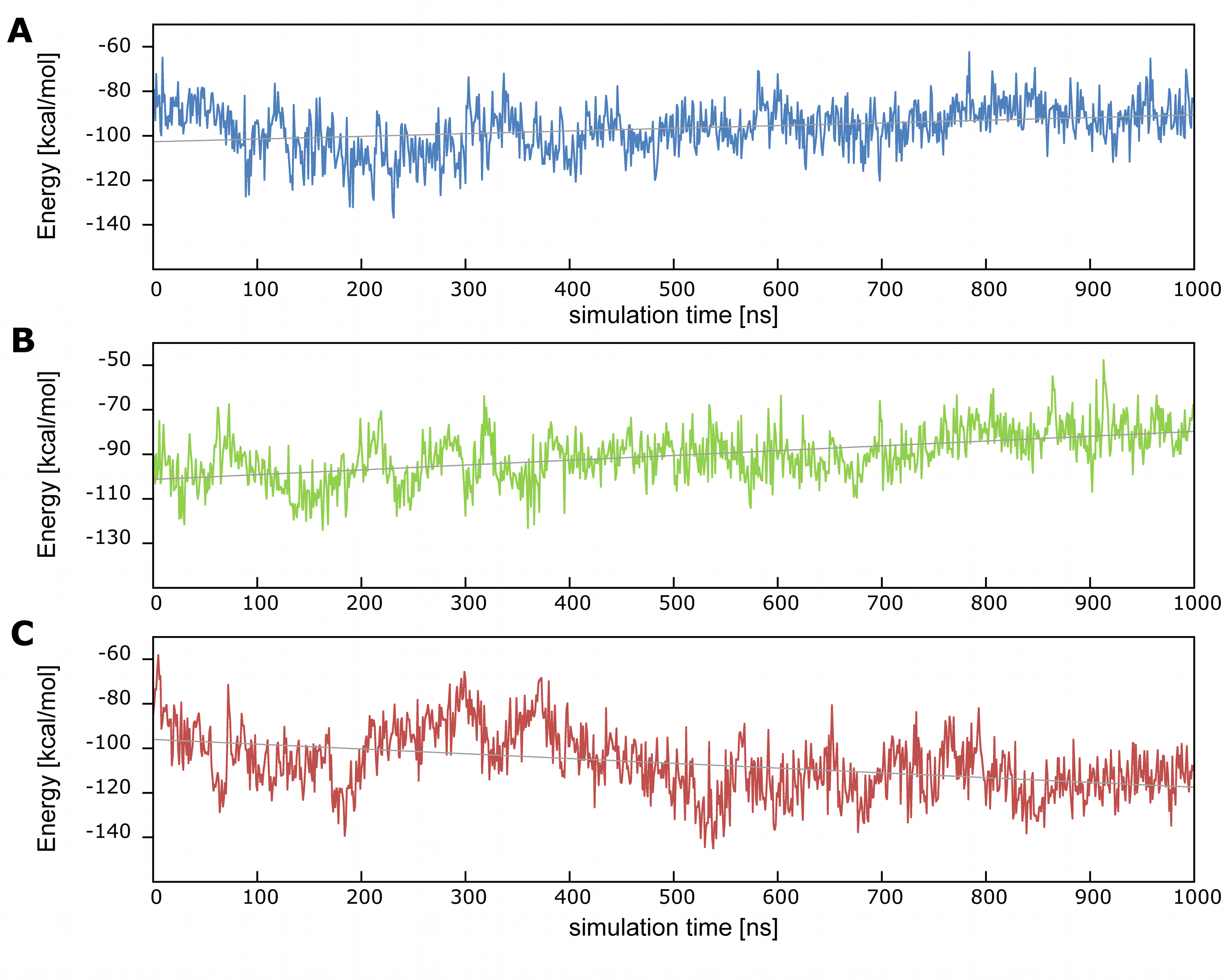

Supplement: Figure S8 — Free energies of binding for the ternary complexes. The free energies of binding for the β2AR-Gαs system (A), for the D2DownR-Gαi system (B) and for the D2UpR-Gαi system (C) are shown. Here, the free energy of binding consists of a molecular mechanics energy term (internal energy of bonds, angles and dihedrals), the polar contribution and the nonpolar contribution of the solvation free energy (polar contribution calculated using the Generalized Born equation and the nonpolar contribution using the molecular solvent-accessible surface area). The curves exhibit a best fit line with a positive gradient for (A) and (B) (0.012 and 0.021 for the β2AR-Gαs- and the D2DownR-Gαi-system, respectively), and a negative gradient for curve (C) (−0.021 for the D2UpR-Gαi-system). As these gradients are very small, we expect that the values will converge to zero for longer simulation times. (TIFF) [file pone.0067244.s008.tiff]

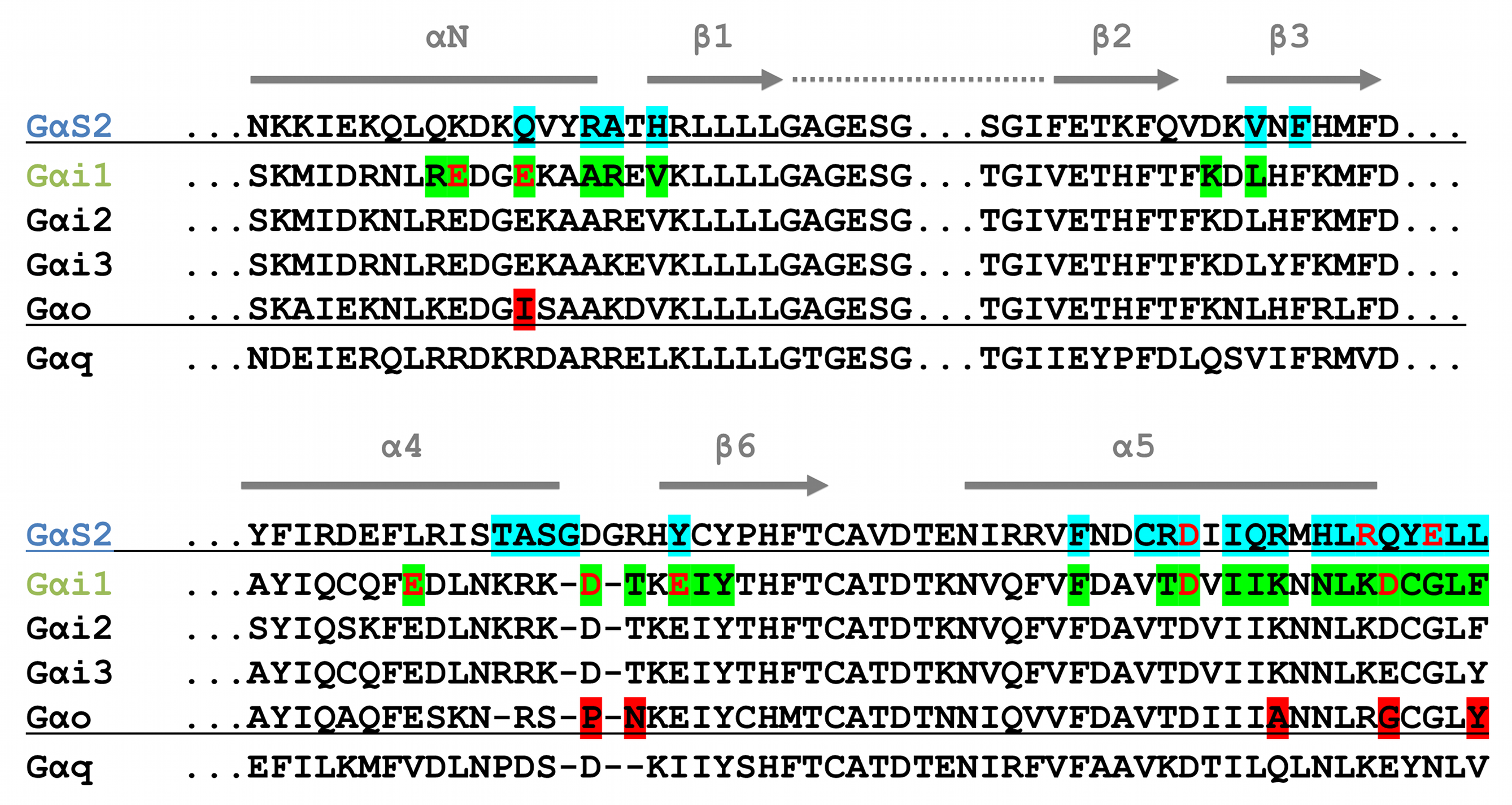

Supplement: Figure S9 — Alignment of contact areas of chosen Gα-subunits. Amino acids within the Gαs and Gαi sequences forming stable contacts to receptor residues are highlighted with a blue and green background, respectively (according to Figure 4). Red backgrounds point to sequence differences between Gαi and Gαo subunits. Red letters indicate residues involved in ionic interactions. (TIFF) [file pone.0067244.s009.tiff]
