# Supplementary material for: Active-State Models of Ternary GPCR Complexes: Determinants of Selective Receptor-G-Protein Coupling
Source: PLoS One. 2013 Jun 24;8(6):e67244. doi: 10.1371/journal.pone.0067244 (PMC3691126; doi:10.1371/journal.pone.0067244)
Supplement: Table S1 — Amino-acid contacts within the β2AR-Gαs-simulation. The occurrence for each amino-acid contact throughout the MD simulation is shown in the grey columns. (DOC) [file pone.0067244.s010.doc]

**Table S1.** Amino-acid contacts within the β2AR-Gα_s_-simulation

|  | **β2AR** | **Gα_s_** |  | **%** |  | **β2AR** | **Gα_s_** |  | **%** |
| --- | --- | --- | --- | --- | --- | --- | --- | --- | --- |
| TM3 | R131 | Y377 | Cterm | 99.98 | TM5 | Q229 | L374 | α5 | 99.44 |
| TM3 | A134 | H373 | α5 | 98.67 | TM5 | Q229 | L380 | Cterm | 99.69 |
| TM3 | A134 | Y377 | Cterm | 70.60 | TM5 | L202 | L380 | Cterm | 99.70 |
| TM3 | I135 | Q370 | α5 | 99.05 | IL3 | K232 | D367 | α5 | 91.06 |
| TM3 | I135 | H373 | α5 | 77.16 | IL3 | K232 | R371 | α5 | 97.17 |
| TM3 | I135 | L374 | α5 | 97.59 | IL3 | I233 | Y344 | β6 | 87.15 |
| TM3 | I135 | Y377 | Cterm | 77.17 | IL3 | I233 | R371 | α5 | 99.23 |
| IL2 | P138 | R366 | α5 | 99.44 | IL3 | I233 | R375 | α5 | 83.46 |
| IL2 | P138 | I369 | α5 | 99.97 | IL3 | I233 | L380 | Cterm | 99.59 |
| IL2 | P138 | Q370 | α5 | 71.84 | IL3 | S236 | T336 | α4 | 96.90 |
| IL2 | F139 | H41 | αNβ1 | 99.90 | IL3 | S236 | A337 | α4 | 76.37 |
| IL2 | F139 | V203 | β2β3 | 99.98 | IL3 | E237 | T336 | α4 | 54.63 |
| IL2 | F139 | F205 | β2β3 | 95.39 | IL3 | E237 | A337 | α4 | 76.78 |
| IL2 | F139 | F362 | α5 | 99.59 | IL3 | E237 | S338 | α4 | 83.34 |
| IL2 | F139 | C365 | α5 | 99.73 | IL3 | E237 | G339 | α4 | 74.80 |
| IL2 | F139 | R366 | α5 | 99.95 | IL3 | E237 | Y344 | β6 | 93.69 |
| IL2 | F139 | I369 | α5 | 88.33 | IL3 | E237 | R375 | α5 | 99.99 |
| IL2 | K140 | V203 | β2β3 | 55.55 | IL3 | G238 | A337 | α4 | 73.47 |
| IL2 | Y141 | H373 | α5 | 92.88 | IL3 | G238 | S338 | α4 | 51.82 |
| IL2 | Q142 | R38 | αNβ1 | 94.73 | IL3 | G238 | G339 | α4 | 52.29 |
| IL2 | S143 | R38 | αNβ1 | 76.32 | TM6 | K270 | R375 | α5 | 51.84 |
| IL2 | S143 | A39 | αNβ1 | 93.82 | TM6 | K270 | E378 | Cterm | 52.77 |
| IL2 | T146 | Q35 | αNβ1 | 52.86 | TM6 | K270 | L379 | Cterm | 50.49 |
| TM5 | V222 | L374 | α5 | 95.63 | TM6 | K270 | L380 | Cterm | 65.63 |
| TM5 | V222 | Y377 | Cterm | 72.00 | TM6 | A271 | L379 | Cterm | 99.91 |
| TM5 | V222 | L379 | Cterm | 99.76 | TM6 | T274 | Y377 | Cterm | 79.88 |
| TM5 | F223 | L379 | Cterm | 82.52 | TM6 | T274 | E378 | Cterm | 88.71 |
| TM5 | E225 | Q370 | α5 | 68.71 | TM6 | T274 | L379 | Cterm | 99.95 |
| TM5 | E225 | L374 | α5 | 87.74 | TM6 | L275 | L379 | Cterm | 57.91 |
| TM5 | A226 | L374 | α5 | 99.91 | TM6 | I278 | Y377 | Cterm | 57.27 |
| TM5 | A226 | L379 | Cterm | 99.89 | TM6 | I278 | L379 | Cterm | 91.68 |
| TM5 | A226 | L380 | Cterm | 76.71 | H8 | P330 | Q376 | Cterm | 97.10 |
| TM5 | Q229 | D367 | α5 | 99.92 | H8 | P330 | E378 | Cterm | 90.10 |
| TM5 | Q229 | Q370 | α5 | 89.73 | H8 | R333 | E378 | Cterm | 92.06 |
| TM5 | Q229 | R371 | α5 | 99.94 |  |  |  |  |  |

The occurrence for each amino-acid contact throughout the MD simulation is shown in the grey columns.
