# Supplementary material for: Active-State Models of Ternary GPCR Complexes: Determinants of Selective Receptor-G-Protein Coupling
Source: PLoS One. 2013 Jun 24;8(6):e67244. doi: 10.1371/journal.pone.0067244 (PMC3691126; doi:10.1371/journal.pone.0067244)
Supplement: Table S2 — Amino-acid contacts within the D2DownR-Gαi-simulation. The occurrence for each amino-acid contact throughout the MD simulation is shown in the grey columns. (DOC) [file pone.0067244.s011.doc]

**Table S2.** Amino-acid contacts within the D2^Down^R-Gα_i_-simulation

|  | **D2^Down^R** | **Gα_i_** |  | **%** |  | **D2^Down^R** | **Gα_i_** |  | **%** |
| --- | --- | --- | --- | --- | --- | --- | --- | --- | --- |
| TM3 | R132 | C351 | Cterm | 99.18 | TM5 | V215 | I344 | α5 | 79.32 |
| TM3 | A135 | N347 | α5 | 99.99 | TM5 | V215 | L348 | α5 | 60.31 |
| TM3 | V136 | I344 | α5 | 99.92 | TM5 | L216 | L348 | α5 | 99.89 |
| TM3 | V136 | N347 | α5 | 68.34 | TM5 | L216 | L353 | Cterm | 91.46 |
| TM3 | V136 | L348 | α5 | 99.89 | TM5 | L216 | F354 | Cterm | 88.16 |
| IL2 | P139 | T340 | α5 | 98.06 | TM5 | R219 | D341 | α5 | 99.95 |
| IL2 | P139 | I343 | α5 | 99.44 | TM5 | R219 | I344 | α5 | 99.66 |
| IL2 | P139 | I344 | α5 | 99.81 | IL3 | R222 | E318 | β6 | 69.90 |
| IL2 | M140 | K192 | β2β3 | 57.01 | IL3 | R222 | I319 | β6 | 57.70 |
| IL2 | M140 | L194 | β2β3 | 98.84 | IL3 | R222 | Y320 | β6 | 86.66 |
| IL2 | M140 | F336 | α5 | 70.74 | IL3 | R222 | D341 | α5 | 66.99 |
| IL2 | M140 | T340 | α5 | 71.67 | IL3 | V223 | E318 | β6 | 77.59 |
| IL2 | Y142 | I343 | α5 | 72.64 | IL3 | K226 | E308 | α4 | 67.13 |
| IL2 | Y142 | N347 | α5 | 97.64 | IL3 | R227 | D315 | α4 | 88.01 |
| IL2 | N143 | A31 | αNβ1 | 70.95 | IL3 | R227 | E318 | β6 | 58.75 |
| IL2 | T144 | E28 | αNβ1 | 86.29 | TM6 | K370 | G352 | Cterm | 95.34 |
| IL2 | T144 | A31 | αNβ1 | 81.46 | TM6 | K370 | L353 | Cterm | 97.24 |
| IL2 | T144 | R32 | αNβ1 | 98.78 | TM6 | K370 | F354 | Cterm | 97.46 |
| IL2 | R145 | E28 | αNβ1 | 80.17 | TM6 | A371 | L353 | Cterm | 99.87 |
| TM4 | S148 | E28 | αNβ1 | 67.62 | TM6 | M374 | L353 | Cterm | 91.21 |
| TM4 | R150 | R24 | αNβ1 | 63.89 | H8 | F429 | D350 | Cterm | 92.73 |
| TM4 | R150 | E25 | αNβ1 | 81.50 | H8 | F429 | C351 | Cterm | 64.25 |
| TM5 | Y209 | L353 | Cterm | 99.26 | H8 | F429 | G352 | Cterm | 89.85 |
| TM5 | I212 | L348 | α5 | 92.48 | H8 | N430 | K349 | α5 | 52.15 |
| TM5 | I212 | L353 | Cterm | 98.26 | H8 | N430 | D350 | Cterm | 56.17 |

The occurrence for each amino-acid contact throughout the MD simulation is shown in the grey columns.
