# Supplementary material for: Active-State Models of Ternary GPCR Complexes: Determinants of Selective Receptor-G-Protein Coupling
Source: PLoS One. 2013 Jun 24;8(6):e67244. doi: 10.1371/journal.pone.0067244 (PMC3691126; doi:10.1371/journal.pone.0067244)
Supplement: Table S3 — Amino-acid contacts within the D2UpR-Gαi-simulation. The occurrence for each amino-acid contact throughout the MD simulation is shown in the grey columns. (DOC) [file pone.0067244.s012.doc]

**Table S3.** Amino-acid contacts within the D2^Up^R-Gα_i_-simulation

|  | **D2^Up^R** | **Gα_i_** |  | **%** |  | **D2^Up^R** | **Gα_i_** |  | **%** |
| --- | --- | --- | --- | --- | --- | --- | --- | --- | --- |
| TM3 | R132 | D350 | Cterm | 99.78 | TM5 | V215 | I344 | α5 | 91.66 |
| TM3 | R132 | C351 | Cterm | 99.49 | TM5 | V215 | L348 | α5 | 57.66 |
| TM3 | A135 | N347 | α5 | 90.20 | TM5 | L216 | L348 | α5 | 99.73 |
| TM3 | A135 | D350 | Cterm | 76.76 | TM5 | L216 | L353 | Cterm | 99.76 |
| TM3 | V136 | I344 | α5 | 99.99 | TM5 | R219 | D341 | α5 | 100.00 |
| TM3 | V136 | N347 | α5 | 98.42 | TM5 | R219 | I344 | α5 | 100.00 |
| TM3 | V136 | L348 | α5 | 99.98 | TM5 | R219 | I345 | α5 | 98.59 |
| TM3 | V136 | C351 | Cterm | 99.84 | TM5 | R219 | L348 | α5 | 96.37 |
| IL2 | P139 | I343 | α5 | 81.60 | IL3 | R222 | Y320 | β6 | 63.12 |
| IL2 | P139 | I344 | α5 | 96.84 | IL3 | V223 | E318 | β6 | 60.59 |
| IL2 | P139 | N347 | α5 | 97.50 | IL3 | V223 | I319 | β6 | 56.17 |
| IL2 | M140 | R32 | αNβ1 | 78.44 | TM6 | Q365 | D315 | α4 | 87.47 |
| IL2 | M140 | V34 | αNβ1 | 62.80 | TM6 | Q366 | D315 | α4 | 68.92 |
| IL2 | M140 | L194 | β2β3 | 98.23 | TM6 | K367 | T316 | α4 | 89.75 |
| IL2 | M140 | I343 | α5 | 89.93 | TM6 | K367 | F354 | Cterm | 98.50 |
| IL2 | N143 | E28 | αNβ1 | 86.45 | TM6 | A371 | L353 | Cterm | 99.71 |
| IL2 | N143 | A31 | αNβ1 | 99.33 | TM6 | M374 | G352 | Cterm | 88.17 |
| IL2 | N143 | R32 | αNβ1 | 73.72 | TM6 | M374 | L353 | Cterm | 99.48 |
| IL2 | T144 | E28 | αNβ1 | 69.17 | TM6 | L375 | L353 | Cterm | 93.52 |
| IL2 | T144 | R32 | αNβ1 | 73.39 | H8 | F429 | K349 | α5 | 54.35 |
| IL2 | R145 | E25 | αNβ1 | 71.99 | H8 | F429 | D350 | Cterm | 90.58 |
| IL2 | R145 | E28 | αNβ1 | 62.59 | H8 | F429 | C351 | Cterm | 99.29 |
| TM4 | S148 | E28 | αNβ1 | 75.14 | H8 | F429 | G352 | Cterm | 96.65 |
| TM4 | R150 | R24 | αNβ1 | 70.58 | H8 | N430 | K349 | α5 | 73.63 |
| TM4 | R150 | E25 | αNβ1 | 83.56 | H8 | N430 | F354 | Cterm | 50.38 |
| TM5 | I212 | C351 | Cterm | 55.74 | H8 | I431 | K349 | α5 | 92.34 |
| TM5 | I212 | L353 | Cterm | 99.89 | H8 | I431 | D350 | Cterm | 92.58 |

The occurrence for each amino-acid contact throughout the MD simulation is shown in the grey columns.
