# Supplementary material for: Active-State Models of Ternary GPCR Complexes: Determinants of Selective Receptor-G-Protein Coupling
Source: PLoS One. 2013 Jun 24;8(6):e67244. doi: 10.1371/journal.pone.0067244 (PMC3691126; doi:10.1371/journal.pone.0067244)
Supplement: Table S4 — Results of the computational alanine scanning for the receptors and the G-proteins. aa refers to the amino acids mutated to alanine. ΔΔG-values are provided in the format ‘value ± standard deviation’. The left column shows the regions within the GPCRs and the G-proteins, to which the mutated amino acids belong. (DOC) [file pone.0067244.s013.doc]

**Table S4.** Results of the computational alanine scanning for the receptors and the G-proteins

|  | **β2AR** | | | **D2^Down^R** | | | **D2^Up^R** | | |
| --- | --- | --- | --- | --- | --- | --- | --- | --- | --- |
|  | **aa** | **ΔΔG (kcal/mol)** | | **aa** | **ΔΔG (kcal/mol)** | | **aa** | **ΔΔG (kcal/mol)** | |
| **TM3**  **-**  **IL2**  **-**  **TM4** | R131A | 2.6 | ± 1.0 | R132A | 2.7 | ± 1.3 | R132A | 9.3 | ± 1.5 |
|  | I135A | 4.1 | ± 1.3 | V136A | 2.3 | ± 0.7 | V136A | 2.2 | ± 0.8 |
|  | P138A | 1.6 | ± 0.6 | P139A | 1.5 | ± 0.5 | P139A | 1.9 | ± 0.5 |
|  | F139A | 9.0 | ± 1.2 | M140A | 2.0 | ± 1.3 | M140A | 4.8 | ± 1.6 |
|  | K140A | -0.4 | ± 1.2 |  |  |  |  |  |  |
|  | Y141A | 1.6 | ± 1.1 | Y142A | 4.4 | ± 1.0 | Y142A | 0.8 | ± 0.4 |
|  | Q142A | 0.2 | ± 1.6 | N143A | -0.3 | ± 0.7 | N143A | 0.8 | ± 1.0 |
|  | S143A | 0.1 | ± 1.0 | T144A | 1.8 | ± 1.0 | T144A | 0.5 | ± 0.6 |
|  |  |  |  | R145A | 1.7 | ± 1.8 | R145A | 7.0 | ± 3.2 |
|  | T146A | 0.2 | ± 0.4 | S148A | 0.3 | ± 0.6 | S148A | 0.5 | ± 1.0 |
|  |  |  |  | R150A | 9.3 | ± 2.5 | R150A | 7.2 | ± 4.0 |
| **TM5**  **-**  **IL3** |  |  |  | Y209A | 1.6 | ± 0.5 | Y209A | 0.2 | ± 0.1 |
|  | V222A | 1.1 | ± 0.6 | I212A | 1.5 | ± 0.6 | I212A | 1.2 | ± 0.5 |
|  | F223A | 0.5 | ± 0.3 |  |  |  |  |  |  |
|  | E225A | 1.9 | ± 2.4 | V215A | 0.4 | ± 0.3 | V215A | 0.5 | ± 0.4 |
|  |  |  |  | L216A | 2.5 | ± 0.8 | L216A | 1.7 | ± 0.5 |
|  | Q229A | 11.1 | ± 3.1 | R219A | 7.7 | ± 2.1 | R219A | 12.3 | ± 1.8 |
|  | L230A | 1.3 | ± 0.7 |  |  |  |  |  |  |
|  | K232A | 3.9 | ± 2.5 | R222A | 11.2 | ± 4.5 | R222A | 2.9 | ± 3.3 |
|  | I233A | 4.6 | ± 1.1 | V223A | 0.6 | ± 0.5 | V223A | 1.0 | ± 1.1 |
|  | S236A | -0.7 | ± 0.6 | K226A | 3.8 | ± 2.0 | K226A | 3.2 | ± 2.4 |
|  | E237A | 16.2 | ± 3.0 | R227A | 11.1 | ± 7.2 | R227A | 3.1 | ± 3.2 |
| **TM6** |  |  |  | Q365A | -0.1 | ± 0.2 | Q365A | 0.3 | ± 1.3 |
|  |  |  |  | Q366A | -0.1 | ± 0.7 | Q366A | 0.3 | ± 0.8 |
|  |  |  |  | K367A | 4.2 | ± 3.5 | K367A | 6.8 | ± 2.2 |
|  | K270A | 4.8 | ± 3.1 | K370A | 6.5 | ± 2.4 | K370A | 0.8 | ± 0.1 |
|  | T274A | 1.3 | ± 1.0 | M374A | 0.5 | ± 0.4 | M374A | 0.7 | ± 0.6 |
|  | L275A | 0.3 | ± 0.2 | L375A | 0.1 | ± 0.1 | L375A | 0.7 | ± 0.4 |
|  | I278A | 1.3 | ± 0.6 |  |  |  |  |  |  |
| **TM7**  **-**  **H8** |  |  |  | F429A | 1.0 | ± 0.6 | F429A | 2.3 | ± 0.7 |
|  |  |  |  | N430A | 0.6 | ± 1.2 | N430A | 2.3 | ± 2.6 |
|  | P330A | 1.7 | ± 0.7 | I431A | -0.1 | ± 0.1 | I431A | 1.6 | ± 0.1 |
|  | R333A | 8.9 | ± 3.2 |  |  |  |  |  |  |

|  | **Gα_s_** | | | **Gα_i_ ^Down^** | | | **Gα_i_ ^Up^** | | |
| --- | --- | --- | --- | --- | --- | --- | --- | --- | --- |
|  | **aa** | **ΔΔG (kcal/mol)** | | **aa** | **ΔΔG (kcal/mol)** | | **aa** | **ΔΔG (kcal/mol)** | |
| **αN**  **-**  **β1** |  |  |  | R24A | -3.7 | ± 0.6 | R24A | -3.3 | ± 0.3 |
|  |  |  |  | E25A | 11.9 | ± 2.6 | E25A | 12.2 | ± 3.9 |
|  | Q35A | 0.7 | ± 1.5 | E28A | 6.8 | ± 2.1 | E28A | 8.1 | ± 4.7 |
|  | R38A | -0.2 | ± 1.3 |  |  |  |  |  |  |
|  |  |  |  | R32A | -2.7 | ± 0.6 | R32A | 2.1 | ± 2.0 |
|  | H41A | 2.6 | ± 1.0 | V34A | -0.1 | ± 0.1 | V34A | 0.1 | ± 0.4 |
| **β2**  **-**  **β3** |  |  |  | K192A | -2.9 | ± 0.3 | K192A | -2.7 | ± 0.2 |
|  | V203A | 1.3 | ± 0.7 | L194A | 1.1 | ± 0.4 | L194A | 1.1 | ± 0.6 |
|  | F205A | 1.1 | ± 0.4 |  |  |  |  |  |  |
|  |  |  |  | E308A | 7.1 | ± 3.0 | E308A | 13.3 | ± 7.1 |
|  | T336A | 1.0 | ± 0.6 |  |  |  |  |  |  |
|  | S338A | -0.1 | ± 0.5 |  |  |  |  |  |  |
|  |  |  |  | D315A | 9.2 | ± 4.0 | D315A | 11.3 | ± 3.2 |
|  |  |  |  | T316A | 0.1 | ± 0.2 | T316A | -0.1 | ± 0.7 |
|  | Y344A | 6.0 | ± 2.0 | E318A | 16.4 | ± 5.2 | E318A | 4.8 | ± 1.5 |
|  |  |  |  | I319A | 0.1 | ± 0.2 | I319A | 0.4 | ± 0.4 |
|  |  |  |  | Y320A | 2.8 | ± 1.1 | Y320A | 2.4 | ± 1.4 |
| **α5** | F362A | 1.4 | ± 0.5 | F336A | 1.3 | ± 0.6 | F336A | 0.1 | ± 0.1 |
|  | C365A | 0.1 | ± 0.2 |  |  |  |  |  |  |
|  | R366A | -1.6 | ± 1.4 | T340A | 0.8 | ± 0.5 | T340A | 0.1 | ± 1.0 |
|  | D367A | 14.6 | ± 4.2 | D341A | 16.5 | ± 6.3 | D341A | 13.5 | ± 2.5 |
|  | I369A | 2.4 | ± 0.8 | I343A | 2.2 | ± 0.7 | I343A | 1.0 | ± 0.5 |
|  | Q370A | 5.3 | ± 2.4 | I344A | 4.0 | ± 0.9 | I344A | 2.6 | ± 1.0 |
|  | R371A | 4.9 | ± 2.5 | K345A | -2.8 | ± 3.3 | K345A | -4.9 | ± 0.9 |
|  | H373A | 2.6 | ± 1.3 | N347A | 4.0 | ± 1.3 | N347A | -0.2 | ± 1.2 |
|  | L374A | 4.9 | ± 0.9 | L348A | 4.2 | ± 1.1 | L348A | 2.9 | ± 0.9 |
|  | R375A | 14.5 | ± 2.2 | K349A | -4.7 | ± 0.8 | K349A | -4.0 | ± 1.0 |
| **C-term.** | Q376A | 0.1 | ± 1.5 | D350A | 1.9 | ± 0.8 | D350A | 6.6 | ± 1.6 |
|  | Y377A | 5.9 | ± 1.2 | C351A | 1.5 | ± 0.8 | C351A | 1.9 | ± 0.8 |
|  | E378A | 9.9 | ± 3.1 |  |  |  |  |  |  |
|  | L379A | 5.8 | ± 1.2 | L353A | 5.4 | ± 1.1 | L353A | 4.5 | ± 1.0 |

aa refers to the amino acids mutated to alanine. ΔΔG-values are provided in the format ‘value ± standard deviation’. The left column shows the regions within the GPCRs and the G-proteins, to which the mutated amino acids belong.
